# Gambogic acid triggers vacuolization-associated cell death in cancer cells via disruption of thiol proteostasis

**DOI:** 10.1038/s41419-019-1360-4

**Published:** 2019-02-22

**Authors:** Min Ji Seo, Dong Min Lee, In Young Kim, Dongjoo Lee, Min-Koo Choi, Joo-Youn Lee, Seok Soon Park, Seong-Yun Jeong, Eun Kyung Choi, Kyeong Sook Choi

**Affiliations:** 1 Department of Biomedical Sciences, Ajou University Graduate School of Medicine, Suwon, 16499 Korea; 2 Department of Biochemistry and Molecular Biology, Ajou University, Suwon, 16499 Korea; 3 Department of Pharmacy, Ajou University, Suwon, 16499 Korea; 4 Department of Pharmacy, Dankook University, Cheonan, 16890 Korea; 5 Chemical Data-Driven Research Center, Korea Research Institute of Chemical Technology, Daejeon, 34114 Korea; 6 Asan Institute for Life Sciences, Department of Convergence Medicine, Asan Medical Center, University of Ulsan College of Medicine, Seoul, 05505 Korea; 7 Center for Advancing Cancer Therapeutics, Department of Radiation Oncology, Asan Medical Center, University of Ulsan College of Medicine, Seoul, 05505 Korea

## Abstract

Gambogic acid (GA), a xanthonoid extracted from the resin of the tree, *Garcinia hanburyi*, was recently shown to exert anticancer activity in multiple studies, but the underlying action mechanism remains unclear. Here, we show that GA induces cancer cell death accompanied by vacuolation in vitro and in vivo. This GA-induced vacuolation in various cancer cells was derived from dilation of the endoplasmic reticulum (ER) and mitochondria, and was blocked by cycloheximide. These findings suggest that GA kills cancer cells by inducing paraptosis, a vacuolization-associated cell death. We found that megamitochondria formation, which arose from the fusion of swollen mitochondria, preceded the fusion of ER-derived vacuoles. GA-induced proteasomal inhibition was found to contribute to the ER dilation and ER stress seen in treated cancer cells, and megamitochondria formation was followed by mitochondrial membrane depolarization. Interestingly, GA-induced paraptosis was effectively blocked by various thiol-containing antioxidants, and this effect was independent of ROS generation. We observed that GA can react with cysteinyl thiol to form Michael adducts, suggesting that the ability of GA to covalently modify the nucleophilic cysteinyl groups of proteins may cause protein misfolding and subsequent accumulation of misfolded proteins within the ER and mitochondria. Collectively, our findings show that disruption of thiol proteostasis and subsequent paraptosis may critically contribute to the anti-cancer effects of GA.

## Introduction

The identification and development of effective anticancer agents with fewer side effects is critical for the successful management of cancer. Gambogic acid (GA) is a xanthone structure isolated from the dry, brownish gamboge resin of *Garcinia hanburyi*. GA has been shown to confer potent anticancer activity via multiple effects in different types of cancers, including inhibition of cancer cell proliferation, induction of apoptosis, inhibition of angiogenesis and inhibition of metastasis^1,2^. Importantly, GA exhibits more toxicity toward cancer cells than to normal cells^1,3,4^. A phase IIb trial of GA is currently being undertaken to test efficacy of GA in treating non-small cell lung, renal and colon cancers^5^.

Malignant cancer cells often show innate and acquired resistance to apoptosis; thus, alternative means to combat malignant cancer via non-apoptotic cell death may offer an attractive therapeutic strategy to effectively kill malignant cancer cells that resist conventional pro-apoptotic cancer therapies. In this study, we show for the first time that GA can kill various cancer cells via the induction of paraptosis. Paraptosis is a programmed cell death mode that is characterized by dilation of the ER and/or mitochondria^6^. It lacks apoptotic features, including chromatin condensation, DNA fragmentation, apoptotic body formation and caspase dependency, and is known to require de novo protein synthesis^6–8^. Recent work has shown that various natural products, including curcumin^9,10^ and celastrol^11^, demonstrate anticancer effects via the induction of paraptosis. Although the molecular basis of paraptosis still remains to be clarified, it is known to be associated with the perturbation of cellular proteostasis via proteasomal inhibition^8–12^ and disruption of sulfhydryl homeostasis^13–16^. In addition, activation of ERKs and JNKs appear to be positively associated with the paraptosis triggered by some inducers, including curcumin^9^, celastrol^11^, and dimethoxycurcumin^12^.

In the present study, we show for the first time that paraptosis is critically associated with the antitumor effect of GA, as shown both in vitro and in vivo. We report that GA-induced formation of megamitochondria due to the mitochondrial swelling/fusion is followed by the fusion of ER-derived vacuoles and the eventual death of cancer cells. Our results show that megamitochondria formation precedes a ROS generation-independent mitochondrial depolarization. And proteasomal inhibition is responsible for GA-induced ER dilation and ER stress. Finally, our data clearly reveal that the ability of GA to covalently modify the sulfhydryl groups of proteins may stress the ER and mitochondria, leading to their dilation and the subsequent cancer cell death.

## Results

### GA induces vacuolation and subsequent cell death in cancer cells

**Fig. 1:**
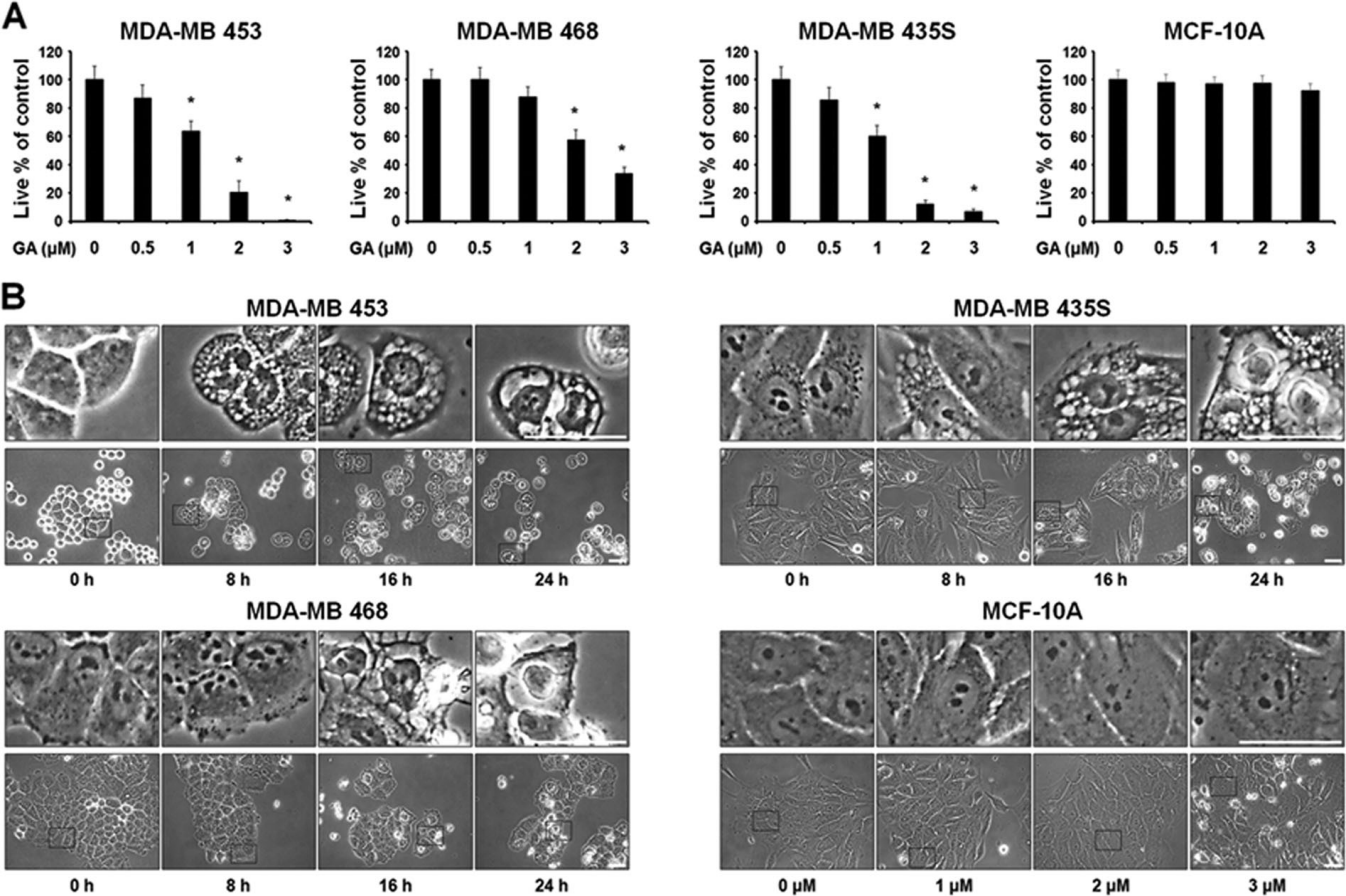

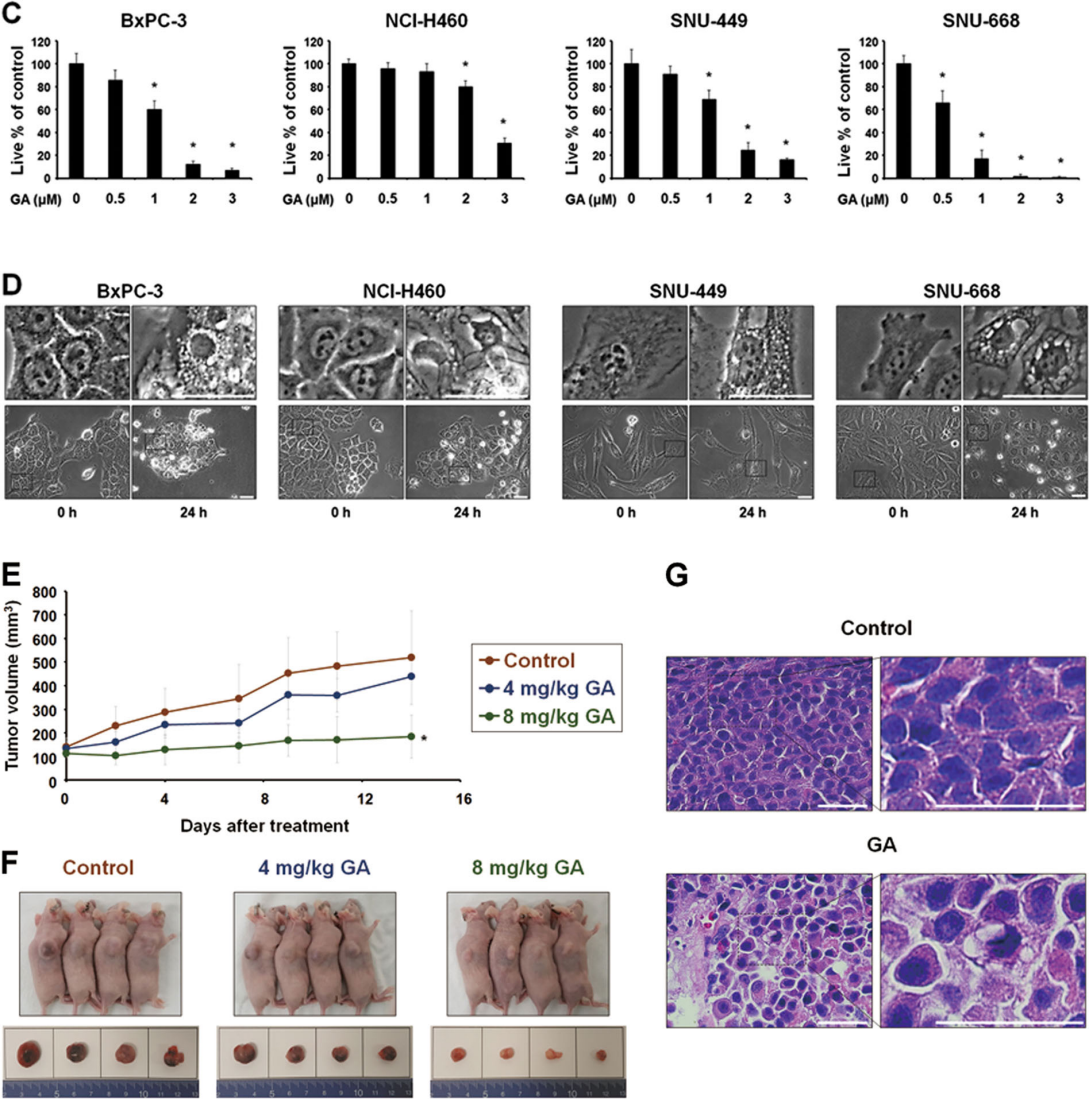
GA induces cell death accompanied by vacuolation in vitro and in vivo. **a**, **c** Cells were treated with the indicated concentrations of GA for 24 h. Cellular viability was assessed using IncuCyte as described in Materials and Methods. Data represent the means ± SEM (*n* = 3). Statistical significance was determined using one-way ANOVA followed by Bonferroni’s *post hoc* tests. *p < 0.01 vs. untreated control. **b**, **d** Cells treated with GA at the concentrations around the IC_50_, (MDA-MB 453 (1.5 μM), MDA-MB 468 (2.35 μM), MDA-MB 435S (1.33 μM), BxPC-3 (1.68 μM), NCI-H460 (2.35 μM), SNU-449 (1.62 μM), and SNU-668 (1.41 μM)), which were calculated using GraphPad Prism, were observed by phase-contrast microscopy. MCF-10A cells treated with GA at the indicated concentrations for 24 h were observed by phase-contrast microscopy. Bars, 40 μm. **e** Athymic nude mice of 6–8 weeks old were xenografted with MDA-MB 435S cells and injected with vehicle, 4 mg/kg GA, and 8 mg/kg GA as described in Materials and Methods. Tumor sizes were measured every 2–3 days after the beginning of vehicle or GA injection and plotted for growth curve. Data represent the means ± SD. Kruskal-Wallis test was performed followed by Dunn’s test. *p *<* 0.05 vs. vehicle-treated mice. **f** treated mice and tumors isolated from those mice were photographed on the 14th day. **g** The results of H&E staining in tumor tissues of the mice treated with 4 mg/kg GA. Bars, 20 μm

The cytotoxic effects of GA were assessed using IncuCyte (Fig. 1a) and Live & Dead kit (Supplementary Fig. 1A), which revealed that GA treatment dose-dependently reduced cell viability in various breast cancer cells, including MDA-MB 453, MDA-MB 468, and MDA-MB 435S cells, but not in human normal counterpart MCF-10A cells. Thus, GA is more cytotoxic to the tested breast cancer cells compared to normal cells. Interestingly, when GA was applied at the IC_50_ dose of each cell line (MDA-MB 453, 1.5 μM; MDA-MB 468, 2.35 μM; and MDA-MB 435S, 1.33 μM) (Supplementary Fig. 1B), a progressive cytoplasmic vacuolation and subsequent cell death were induced; in contrast, MCF-10A cells were relatively resistant to this effect of GA at doses up to 3 μM (Fig. 1b). When we tested the effect of GA on other types of cancer cells, we found that it induced similar cellular responses in BxPC-3 (pancreatic cancer), NCI-H460 (lung cancer), SNU-449 (hepatocellular carcinoma), and SNU-668 (gastric cancer) cells (Figs. 1c, d). These results suggest that GA induces vacuolation and subsequent cell death in various cancer cells. Next, we evaluated the anticancer effect of GA in vivo using a MDA-MB 435S cell xenograft model. Mice received intraperitoneal (i.p.) injections of saline or GA (4 or 8 mg/kg) twice (day 0 and day 2). Tumor volume and body weight was measured three times a week until the 14th day after the beginning of injection. We found that GA dose-dependently reduced the tumor size (Figs. 1e, f) without inducing any significant loss of body weight (Supplementary Fig. 2). Hematoxylin and eosin (H&E) staining showed that cellular vacuolation was present in MDA-MB 435S xenograft sections obtained from GA-treated mice (Fig. 1g). Collectively, these results indicate that GA demonstrates an anticancer effect via the induction of cell death accompanied by vacuolation both in vitro and in vivo.

### GA induces paraptosis in cancer cells

To investigate which cell death pathway is critically involved in the anticancer effect of GA, we used z-VAD-fmk (an apoptosis inhibitor), necrostatin-1 (a necroptosis inhibitor), 3-methyladenine (an early-phase autophagy inhibitor) and bafilomycin A1 (a late-phase autophagy inhibitor). However, the tested inhibitors did not significantly affect the GA-induced cell death and vacuolation of various cancer cell lines (Supplementary Fig. 3), suggesting that apoptosis, necroptosis, and autophagy may not play critical roles in the GA-induced anticancer effect in these cancer cells.

**Fig. 2:**
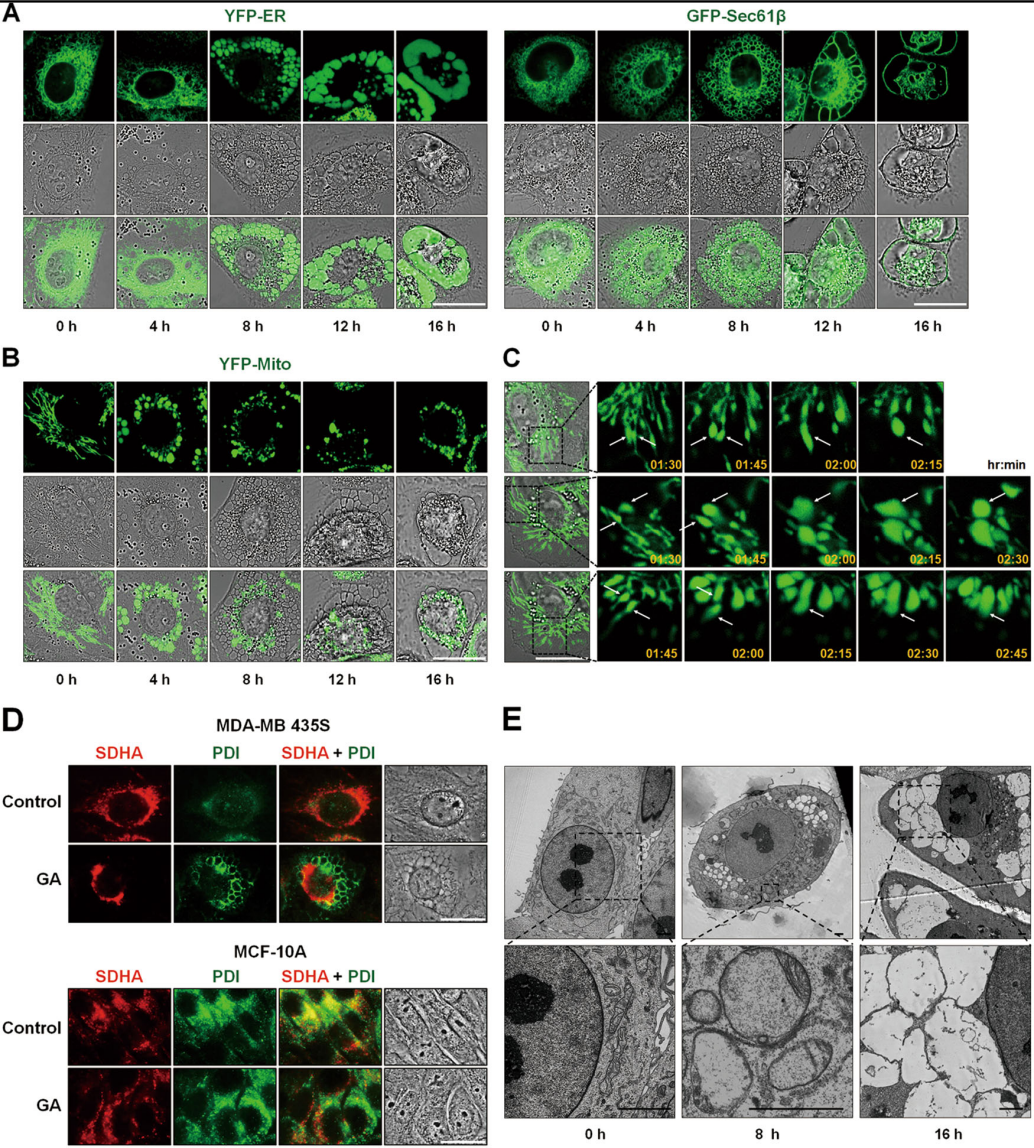
GA induces the paraptotic morphologies in cancer cells. **a**, **b** YFP-ER or GFP-Sec61β cells (**a**) and YFP-Mito cells (**b**) and treated with 1 μM GA for the indicated time points were observed under the confocal microscope. Representative pictures of cells are shown. Bars, 20 μm. **c** Time-lapse imaging results of YFP-Mito cells treated with GA under the confocal microscope. Representative pictures of cells are shown. Bars, 20 μm. **d** Cells were treated with 1 μM GA for 12 h, fixed, and subjected to the immunocytochemistry of PDI and SDHA. Bars, 20 μm. **e** Transmission electron microscopy of MDA-MB 435S cells treated with 1 μM GA for 24 h. Bars, 2 μm

To further evaluate the cell death mode induced by GA in our tested cancer cells, we examined the origins of the GA-induced vacuoles. Since autophagy inhibitors did not affect GA-induced vacuolation, we investigated the possible involvement of mitochondria and/or the ER in this process. Experiments using the YFP-ER plasmid, which labels the ER lumen, and the GFP-Sec61β plasmid, which labels the ER membrane, showed that reticular ER structures were observed in cells treated with GA for 4 h (Fig. 2a). In contrast, we observed ring-shaped fluorescence in both YFP-ER (within the ER lumen) and GFP-Sec61β (around the ER vacuole) cells at 8 h or GA treatment. Thereafter, the dilated ER progressively fused until most of cellular spaces (except the nucleus) were occupied by dramatically expanded ER vacuoles. Analysis employing the YFP-Mito plasmid, which labels the mitochondrial matrix, revealed that elongated and filamentous mitochondrial morphologies were detected in untreated cells (Fig. 2b). In contrast, large mitochondria-derived vacuoles were observed around the nuclei of cells treated with 1 μM GA for 4 h, and slightly smaller, but still enlarged mitochondria were observed at later time points. Our time-lapse imaging revealed that GA initially triggered mitochondrial swelling, which was followed by the fusion of swollen mitochondria, leading to the formation of giant mitochondria (megamitochondria) with oval or spherical shape (Fig. 2c). Immunocytochemistry showed that the expression of succinate dehydrogenase (SDHA, an inner mitochondrial membrane protein) was detected as a small and filled ring shape at the perinuclear area, while the expression of protein disulfide isomerase (PDI, an ER-resident protein) was observed as a larger ring shape at the cellular periphery in MDA-MB 435S cells (Fig. 2d). In contrast, we did not observe any noticeable alteration in the mitochondria or ER of MCF-10A cells treated with GA. Electron microscopy further revealed that megamitochondria and slightly expanded ER structures were observed in MDA-MB 435S cells treated with 2 μM GA for 8 h, whereas lengthy and filamentous mitochondria and regular ER structures were detected in untreated cells (Fig. 2e). At 16 h of GA treatment, most of the cellular spaces were occupied by ER-derived vacuoles undergoing fusion. Taken together, these results indicate that GA induces the morphological features of paraptosis, a cell death mode accompanied by dilatation of mitochondria and the ER.

**Fig. 3:**
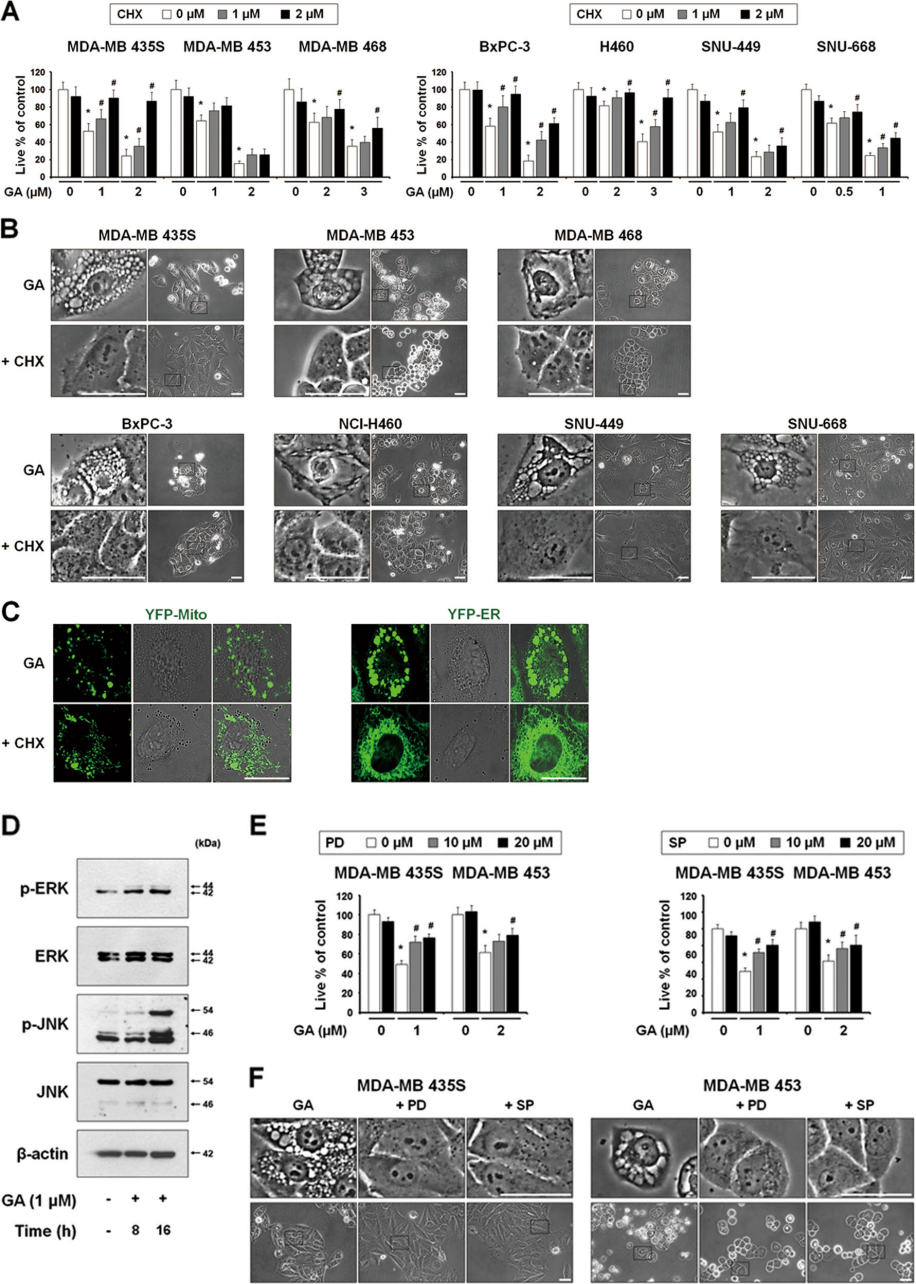
(See legend on next page.)

Accordingly, we examined whether GA induces the biochemical features of paraptosis. Although the molecular basis of paraptosis still remains to be clarified, it is known to require de novo protein synthesis^6^ and to be commonly characterized by the induction of ER stress^8^. We tested the effect of pretreatment with the protein synthesis blocker, cycloheximide (CHX), and found that it very effectively blocked GA-induced cell death and vacuolation in all of the tested cancer cell lines (Figs. 3a, b). The GA-induced mitochondrial dilation observed in YFP-Mito cells and the ER dilation observed in YFP-ER cells were also markedly blocked by CHX pretreatment (Fig. 3c). Since MAP kinases, including ERKs and JNKs, have been positively associated with paraptosis^7–9,11,12^, we next investigated whether GA activates these kinases. In cells treated with 1 μM GA, the phosphorylation levels of ERKs and JNKs were markedly increased (Fig. 3d). In addition, pretreatment of MDA-MB 435S and MDA-MB 453 cells with either PD98059, a MEK inhibitor, or SP600125, a JNK inhibitor, partially but significantly attenuated GA-induced cell death and vacuolation (Figs. 3e, f). Taken together, these results indicate that GA-induced cell death in the tested cancer cells shares the biochemical features of paraptosis.

### GA induces ER stress due to proteasomal inhibition and triggers mitochondrial depolarization without ROS generation

**Fig. 4:**
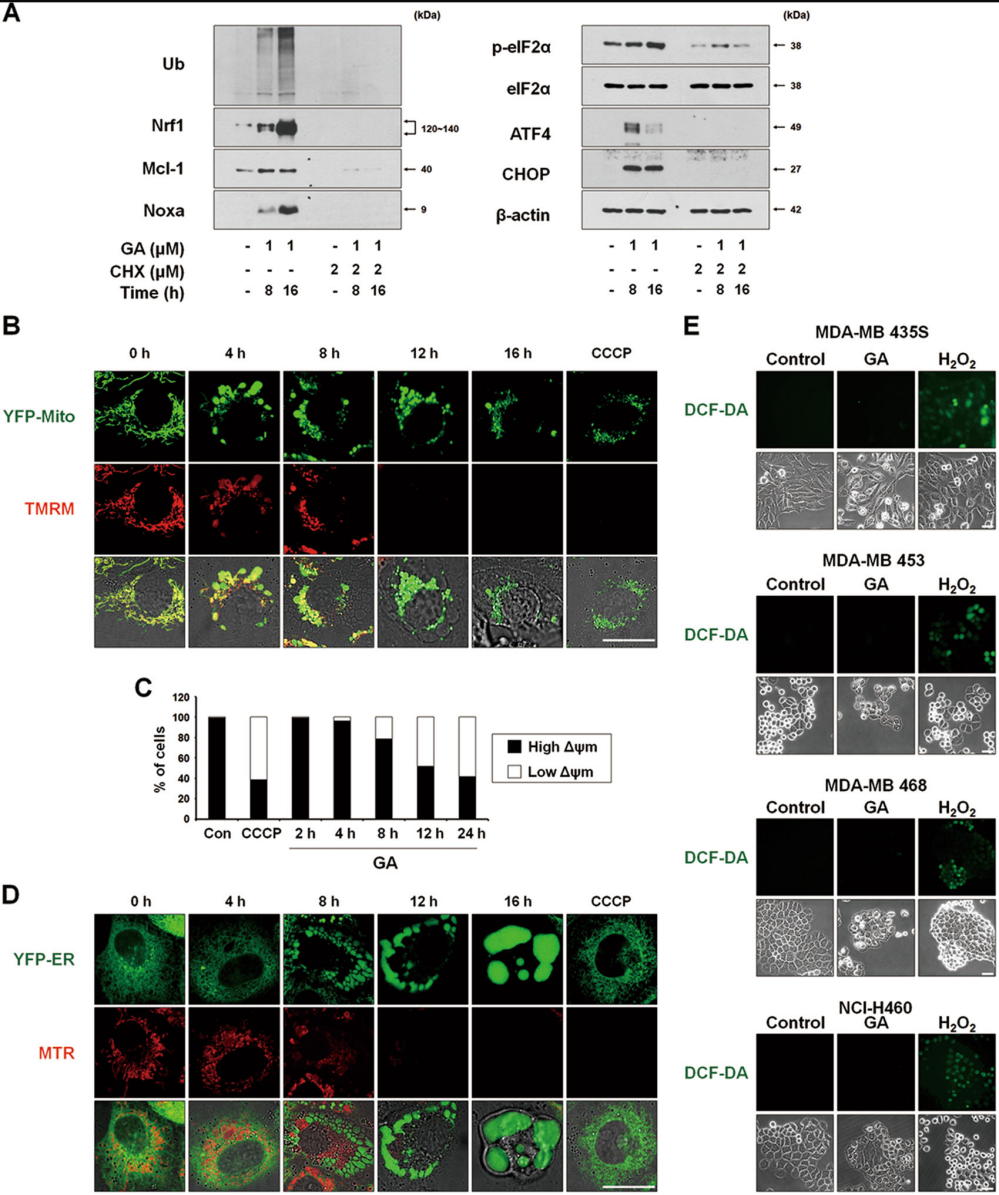
ROS levels are not noticeably increased in spite of the MMP loss in GA-induced paraptosis. **a** MDA-MB 435S cells pretreated with or without 2 μM CHX and further treated with 1 μM GA for the indicated time points. Cell extracts were prepared for Western blotting of the indicated proteins. Western blotting of β-actin was used as a loading control. **b, c** YFP-Mito cells (**b**) or MDA-MB 435S cells (**c**) were treated with 1 μM GA for the indicated time points or 20 μM CCCP for 12 h were incubated with TMRM. Samples were subjected for confocal microscopy (**b**) or flow cytometry (**c**). **d** YFP-ER cells were treated with 1 μM GA for the indicated time points or 20 μM CCCP for 12 h. Treated cells were incubated with MTR and observed under the confocal microscope. **e** Cells treated with GA (1 μM for MDA-MB 435S; 2 μM for MDA-MB 453; 3 μM for MDA-MB 468 and NCI-H460 cells) for 16 h or 5 mM H_2_O_2_ for 10 min were incubated with CM-H_2_DCF-DA (DCF-DA) and subjected for the fluorescence microscopy. Bars, 40 μm

We previously showed that proteasome inhibition-triggered proteostatic disruption critically contributes to paraptosis, particularly in the contexts of ER stress and ER dilation^8,9,12^. Thus, to investigate the mechanism underlying GA-induced paraptosis, we tested whether GA affects proteasome activity. We found that GA treatment progressively increased the levels of poly-ubiquitinated proteins, proteasome-substrate proteins (e.g., Nrf1, Mcl-1, and Noxa) and ER stress marker proteins (e.g., phospho-eIF2α, ATF4, and CHOP) (Fig. 4a). CHX pretreatment effectively blocked the GA-induced accumulation of the tested proteasome-substrate proteins and ER stress marker proteins (Fig. 4a). Taken together, these results indicate that GA induces paraptosis in various cancer cells, and that proteasome inhibition may be closely associated with the ER stress and ER dilation seen during this process.

To investigate how GA affects mitochondrial function during paraptosis, we analyzed the mitochondrial membrane potential (MMP, Δ*ψ*) using tetramethylrhodamine methyl ester (TMRM). Interestingly, although the MMP was not markedly altered in the megamitochondria of YFP-Mito cells treated with GA for 4 h, it was lost in those subsequently undergoing mitochondrial fragmentation (Figs. 4b, c and Supplementary Fig. 4). Staining of YFP-ER cells with MitoTracker-Red (MTR) showed that this MMP loss was accompanied by a marked expansion of the ER-derived vacuoles (Fig. 4d). The loss of MMP after exposure to various stimuli is often followed by the production of reactive oxygen species (ROS)^17^, and several studies showed that GA-induced ROS play a critical role in its anticancer effects^2,18^. Therefore, we tested the possible involvement of ROS in GA-induced paraptosis. However, measurement of ROS using CM-H_2_DCF-DA revealed that GA treatment did not noticeably increase the levels of ROS in the tested cancer cells, whereas H_2_O_2_ treatment (positive control) did trigger such a response (Fig. 4e and Supplementary Fig. 5).

### Thiol-containing antioxidants effectively block GA-induced paraptosis, independently of ROS generation

**Fig. 5:**
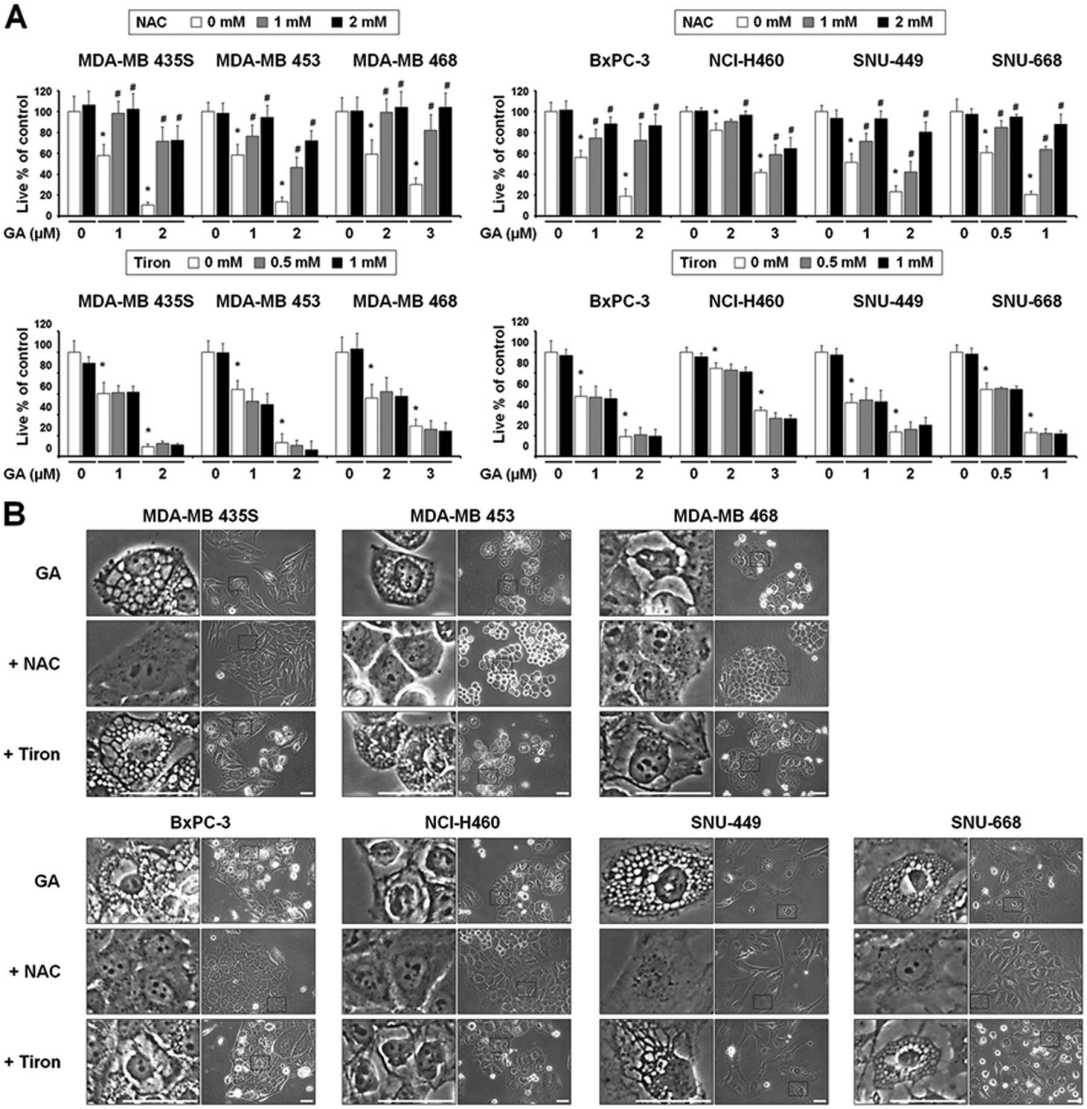

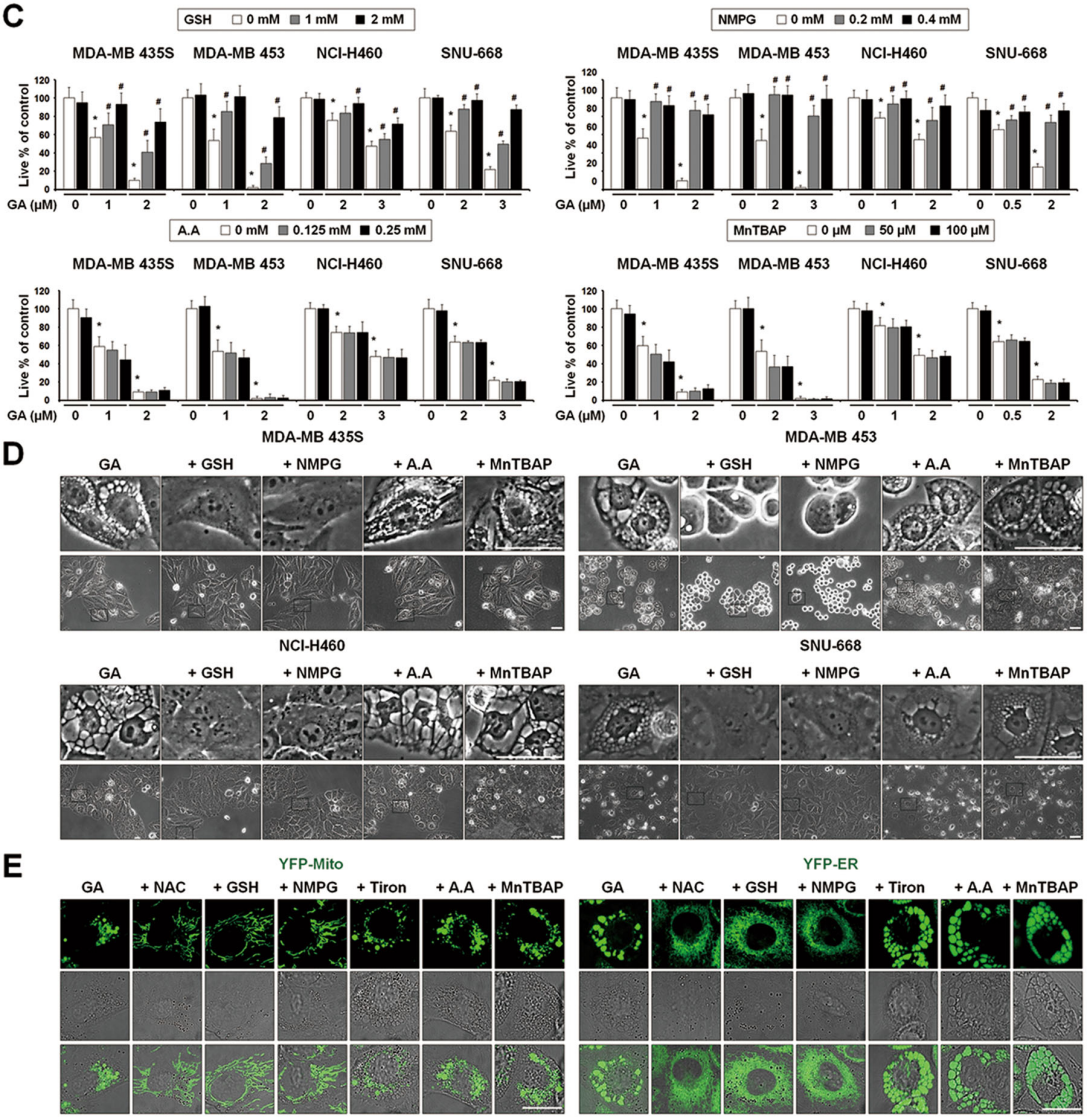
(See legend on next page.)

To confirm that ROS are not involved in GA-induced paraptosis, we tested the effects of various antioxidants. Interestingly, the general antioxidant, *N*-acetylcysteine (NAC), but not the mitochondria-targeted antioxidant, Tiron^19^, dose-dependently blocked GA-induced cell death and vacuolation in all of the tested cancer cell lines (Figs. 5a, b). When we further examined the effects of other antioxidants on GA-induced cellular responses, we found that thiol-containing antioxidants, including glutathione (GSH), and *N*-(2-mercapto-propionyl)-glycine (NMPG), but not non-thiol ROS scavengers, such as, ascorbic acid (Vitamin C) and manganese (III) tetrakis (4-benzoic acid) porphyrin chloride (MnTBAP; a superoxide dismutase mimetic), also very effectively blocked GA-induced cell death in all of the tested cancer cells (Fig. 5c). Moreover, the GA-induced cellular vacuolation in these cells (Fig. 5d), the dilation of the ER in YFP-ER cells and the dilation of mitochondria in YFP-Mito cells (Fig. 5e) were effectively blocked by various thiol antioxidants but not by non-thiol antioxidants. Collectively, these results suggest that thiol-antioxidants block GA-induced paraptosis in a ROS-independent manner.

### The formation of an adduct between GA and thiol-containing proteins may critically contribute to GA-induced paraptosis

**Fig. 6:**
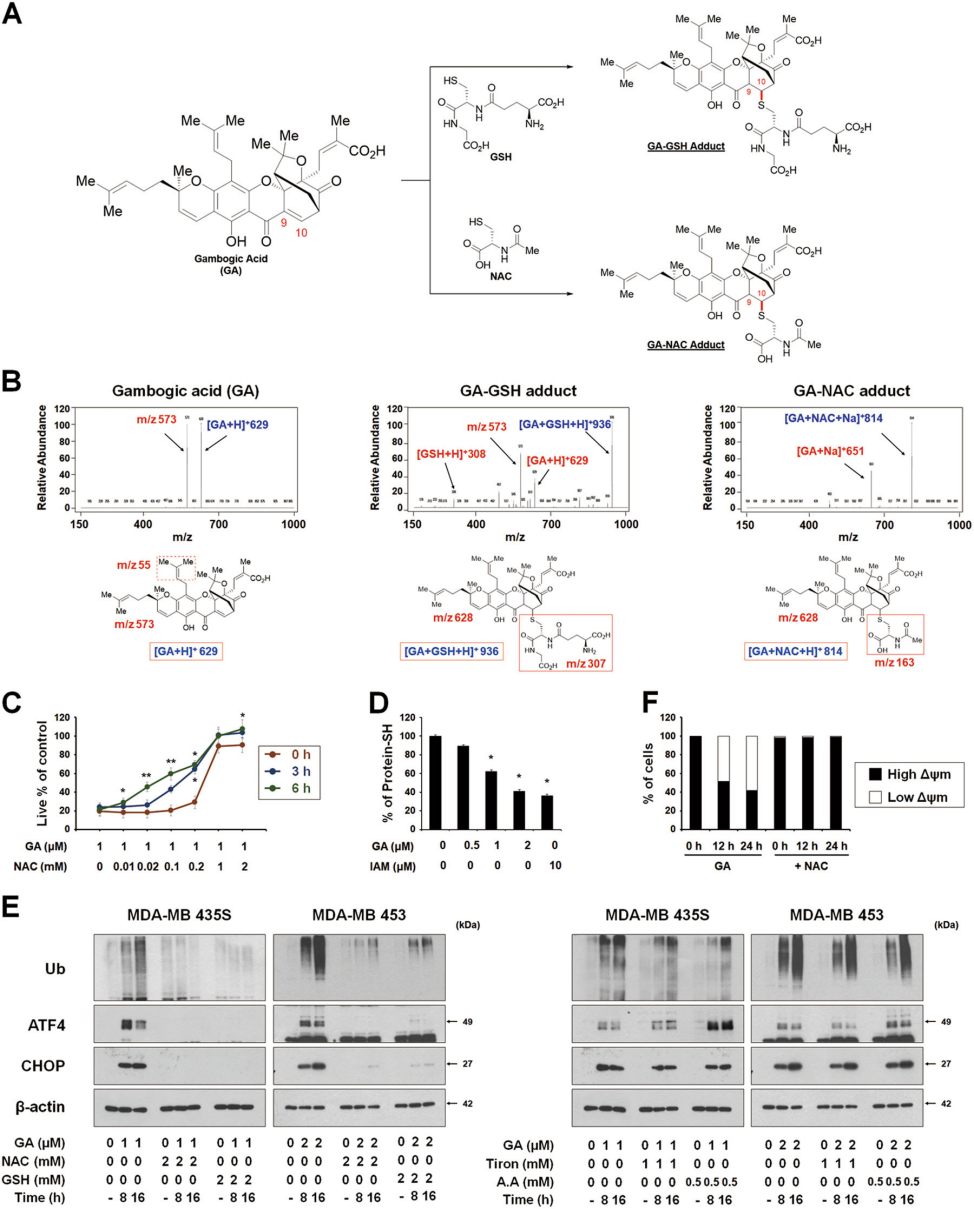
The activity of GA to bind to thiol-containing proteins may be critical for its paraptosis-induced ability in cancer cells. **a** Proposed chemical structures of the GA-GSH and GA-NAC adducts. **b** Full-scan product ion scan spectra and the expected structures of GA, GA-GSH, and GA-NAC adduct formed upon Michael addition of GSH or NAC. The *m/z* values of the GA-GSH adduct represent GSH at 308, GA at 629, and the adduct form at 936. The *m/z* values of the GA-NAC adduct represent NAC at 164, GA at 651, and the adduct form at 814. **c** Increasing concentrations of NAC were pre-incubated with 1 μM GA in serum-free medium for the indicated time durations at room temperature, and these mixtures were used to treat MDA-MB 435S cells for 24 h. The cell viability was measured using IncuCyte. Data represent the means ± SD. Kruskal-Wallis test was performed followed by Dunn’s test. *p < 0.05; **p < 0.01 vs. GA-treated cells. **d** MDA-MB 435S cells were treated with the indicated concentrations of GA or 10 μM iodoacetamide (IAM; a positive control to reduce intracellular protein-SH levels) for 12 h. Protein-SH levels were measured using the dibromobimane (dBrB) assay, as described in the Materials and Methods. Data represent the means ± SD. Kruskal-Wallis test was performed followed by Dunn’s test. *p < 0.01 vs. untreated control. **e** Cell extracts were prepared from the cells pretreated with the antioxidants and further treated with GA (1 μM for MDA-MB 435S and 2 μM for MDA-MB 453 cells) for the indicated concentrations for Western blotting. β-actin was used as a loading control. **f** MDA-MB 435S cells pretreated with 2 mM NAC and further treated with 1 μM GA for the indicated time points were incubated with TMRM. Samples were subjected for flow cytometry

**Fig. 7:**
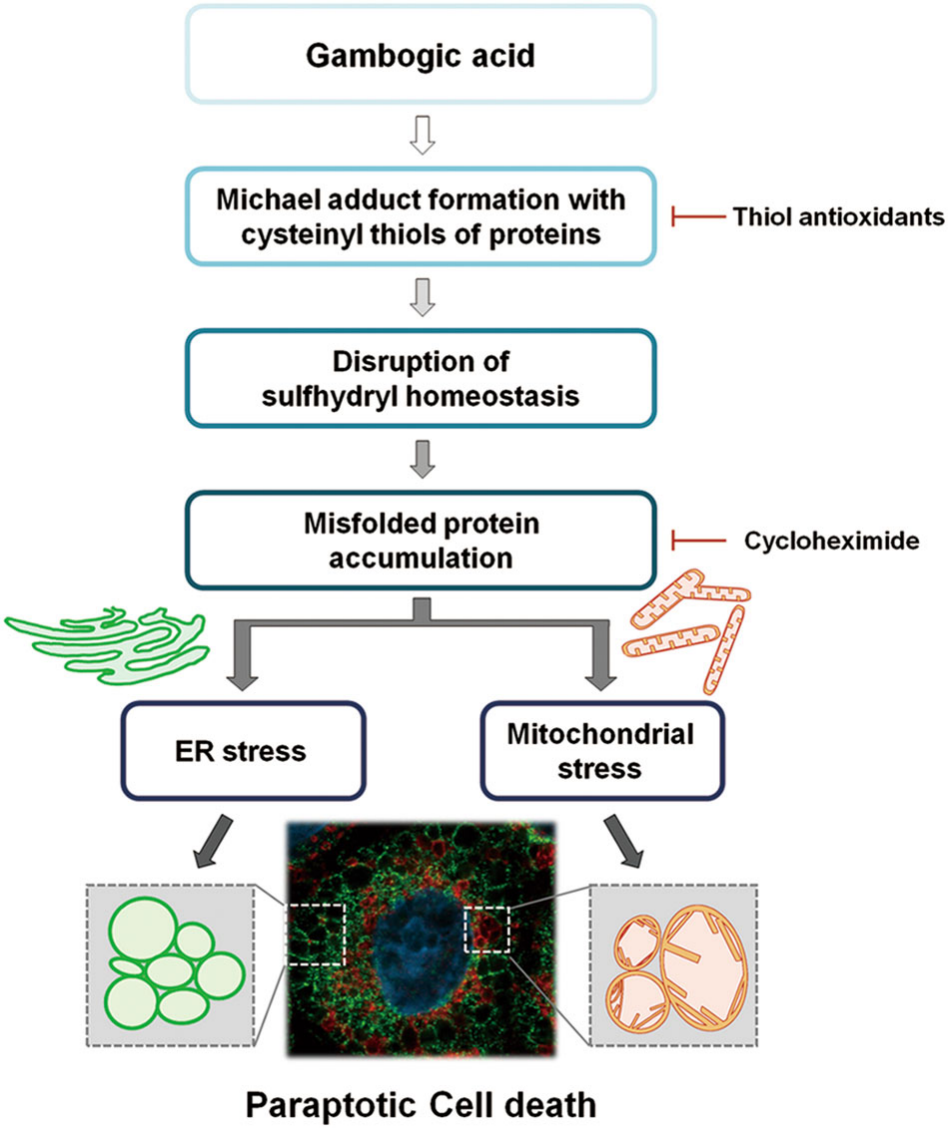
Hypothetical model for the underlying mechanisms of GA-induced paraptosis

Considering the highly electrophilic character of the α,β-unsaturated ketone substructure of GA at C10^20,21^, we examined the possibility that GA may react with the thiol groups of GSH and NAC to form covalent Michael adducts, such as GA-GSH and GA-NAC conjugates (Fig. 6a). To test this hypothesis, we incubated 100 μM GA with excess 50 mM GSH for 3 h and then analyzed the reaction mixture by LC-MS/MS. The MS scan revealed a peak at *m/z* 936 ([M + H]^+^), which corresponded to the molecular weight of the Michael addition product, and its representative fragmentation pattern in the product ion scan showed peaks at *m/z* 629 ([M + H]^+^) and 308 ([M + H]^+^), which corresponded to the molecular weights of GA and GSH, respectively (Fig. 6b). The LC-MS/MS chromatogram obtained after incubation of 100 μM GA with 50 mM NAC showed the GA-NAC adduct peak at *m/z* 814 ([M + Na]^+^), confirming that an adduct was formed between GA and NAC. To further examine whether the interaction between GA and NAC affected GA-mediated cytotoxicity, we pre-incubated cells with 1 μM GA plus different doses of NAC in serum-free medium at room temperature for different durations to allow the formation of chemical adducts, and then treated MDA-MB 435S cells with each GA-NAC mixture for 24 h. At a given dose of NAC, cells treated with GA and NAC that had undergone the prolonged pre-incubation showed far less GA-mediated cytotoxicity than those subjected to simultaneous treatment; moreover, pre-incubation required a lower concentration of NAC to block GA-mediated cell death to the same extent, compared to simultaneous treatment (Fig. 6c). These results strongly suggest that NAC blocks GA-induced cytotoxicity by eliminating its ability to form Michael adducts, particularly with the nucleophilic thiol groups of intracellular proteins. To further test whether GA directly reacts with the free thiol residues of proteins, we performed the dibromobimane (dBrB) assay, which is based on the ability of dBrB to react with free reduced thiols and generate a highly fluorescent protein-dBrB adduct^22,23^. We used iodoacetamide (IAM), an alkylating agent that reacts with protein-SH groups to form stable S-carboxyaminodimethyl-cysteine adducts^23,24^, as a positive control. Indeed, IAM treatment effectively reduced the free protein-SH levels in MDA-MB 435S cells (Fig. 6d). Importantly, GA treatment also dose-dependently decreased the protein-SH levels in these cells, suggesting that stable adducts formed between GA and thiol-containing proteins to disrupt intracellular thiol homeostasis. Supporting this idea, the GA-induced accumulations of poly-ubiquitinated proteins, phospho-eIF2α, ATF4 and CHOP were effectively inhibited only by thiol antioxidants (Fig. 6e). In addition, the GA-induced loss of MMP was almost completely blocked by NAC treatment (Fig. 6f). Taken together, our results suggest that the GA-induced covalent modification of the free thiol groups of intracellular proteins may interfere with proper disulfide bond formation during protein folding and induce the accumulation of misfolded proteins within the ER and mitochondria, leading to stress and dilation of these organelles, and eventual paraptotic cell death (Fig. 7).

## Discussion

GA has been shown to demonstrate anticancer effects by inhibiting the cell growth of various types of human cancer cells in vitro and in vivo^3,25–28^, by inducing apoptosis^3,25,26,29^, and by inhibiting metastasis and angiogenesis^30,31^. Several in vivo studies on adults of different animal species have demonstrated that GA exhibits a good safety profile^32^. It has also been shown to enhance the cytotoxic effects of various chemotherapeutic agents, such as docetaxel^33^, oxaliplatin^34^, adriamycin^35^, imatinib^3^, and bortezomib^36^, in various cancer cells, while reducing the side effects associated with these agents. Thus, GA appears to have great potential as an effective agent for the prevention and treatment of different cancers. However, despite its potent anticancer efficacy, the molecular mechanisms underlying the actions of GA remain still elusive. In this study, we show for the first time that GA can induce antitumor effects by inducing paraptosis as a major cell death mode. In the tested cancer cells, GA treatment rarely yielded apoptotic morphologies and the GA-induced anticancer effect was not significantly dependent on caspases. Given the problems with malignant cancer cells showing genetic heterogeneity as well as innate and acquired resistance to apoptosis^37,38^, it may be useful to apply a strategy to induce an alternative cancer cell death mode whose regulatory mechanisms differ from those of apoptosis. Paraptosis is such a cell death mode, and its induction may greatly contribute to improving cancer therapy. We observed extensive vacuolation and subsequent cell death in various types of cancer cells exposed to GA and in MDA-MB 435S xenograft sections obtained from GA-treated mice. This GA-induced vacuolation originated from the ER and mitochondria, as assessed using relevant expression plasmids (YFP-ER, GFP-Sec61β and YFP-Mito), immunocytochemical analysis of changes in the ER or mitochondria, and electron microscopy. In response to GA, megamitochondria formed due to the swelling and fusion of mitochondria; this was followed by the expansion of ER-fusion-derived vacuoles. Pretreatment with CHX, which is known as an inhibitor of paraptosis^6^, very effectively blocked the dilation of the ER and mitochondria and subsequent cell death in the tested cancer cell lines.

If we hope to deconvolute the mechanisms of action underlying the biological or therapeutic action of GA, we must understand its molecular targets. GA has been shown to directly bind and inhibit various functionally important proteins, including the proteasome^39^, Hsp90β^40^, IKKβ^41^, topoisomerase IIα^42^, transferrin receptor^43^, thioredoxin 1/2^44^, MDM2^45^, and XPO2^46^. However, the primary cellular target(s) and action mode(s) of GA remain unclear. It is also interesting to consider how GA can act as an effective multi-targeted agent. The 9,10-carbon double bond of the α,β-unsaturated ketone of GA was proposed to be imperative for its cytotoxicity^20,21,29^ and has been shown to directly interact with the cysteine residues of IKKβ^41^, thioredoxin 1/2^44^, and XPO2^46^. Here, we propose that the 9,10-carbon double bond of the α,β-unsaturated ketone of GA may act as an electrophilic Michael addition center to covalently modify nucleophilic cysteinyl thiols of its multiple target proteins to trigger paraptosis. This hypothesis is supported by the following findings presented herein: (a) GA undergoes chemical reactions with GSH and NAC via their thiol nucleophilic thiol structures to generate GA-GSH and GA-NAC adducts, respectively, in vitro. (b) Pre-incubation of NAC with GA ameliorates the cytotoxicity of GA toward MBA-MB 435S cells. (c) Various thiol antioxidants block all of the tested GA-induced cellular responses, including proteasome inhibition, ER stress, ER dilation, mitochondrial dilation and MMP loss, leading to cell death in cancer cells of different tissue origins. (d) GA treatment reduces the intracellular sulfhydryl protein (protein-SH) content in MDA-MB 435S cells, suggesting that the formation of Michael adducts with intracellular cysteinyl thiols of proteins may critically contribute to GA-induced toxicity. Given this, we might next ask: What is the underlying mechanism by which GA triggers dilation of the ER and mitochondria? One possible mechanism is that the electrophilic nature of GA may disrupt proper disulfide bond formation to cause misfolding of the most accessible proteins, leading to accumulation of misfolded proteins within the ER and mitochondria. In particular, GA-mediated binding and subsequent inhibition of the machineries associated with proteostasis, such as the proteasome, may result in a dramatic accumulation of misfolded proteins in the cell. Previously, Mimnaugh et al.^47^ proposed that a proteasome-inhibition-triggered overload of misfolded proteins in the ER lumen could exert an osmotic pressure that draws water from the cytoplasm to distend the ER lumen. In the present study, we found that GA inhibited proteasome activity, as evidenced by the accumulation of poly-ubiquitinated proteins and proteasome-substrate proteins. Inhibition of de novo protein synthesis by CHX pretreatment blocked this GA-mediated response, possibly reducing the protein load in the ER. Accumulation of misfolded proteins within the mitochondria has also been proposed to lead to mitochondrial stress and dilation^48,49^, in a manner similar to ER dilation^50^. However, the exact cause of mitochondrial dilation remains to be clarified. Ultimately, the physical and functional perturbation of the two organelles may lead to MMP loss and irreversible paraptotic cell death. Regarding the potential targets through which GA could contribute to paraptosis, we speculated that MDM2 may be involved. GA has been reported as a putative MDM inhibitor^45^ and a fraction of MDM2 was recently shown to be imported to control mitochondrial dynamics independently of p53^51^. In addition, we recently reported that the MDM2 inhibitor, nutlin-3, induces mitochondrial dilation^49^. Furthermore, combination treatment of MDA-MB 435S cells with nutlin-3 and proteasome inhibitors (e.g., bortezomib) effectively induced paraptotic cell death^49^. Based on the present and previous results, we hypothesized that GA may induce paraptosis via dual inhibition of the proteasome and MDM2. To address this, we first tested whether GA-mediated paraptosis can be sensitized by inhibition of the proteasome or MDM2. We found that low doses of GA (up to 0.5 μM), bortezomib (up to 10 nM) or nutlin-3 (up to 40 μM) did not noticeably induce cellular vacuolation and cell death (Supplementary Fig. 6A,B). However, bortezomib or nutlin-3 dose-dependently enhanced vacuolation and cell death in MDA-MB 435S cells treated with 0.5 μM GA, similar to the effect of bortezomib plus nutlin-3 (Supplementary Fig. 6A, B). In addition, 5 nM bortezomib plus 0.5 μM GA or 30 μM nutlin-3 plus 0.5 μM GA markedly induced the dilation of both the ER and mitochondria, similar to the effect of bortezomib plus nutlin-3 (Supplementary Fig. 6C). These observations begin to suggest that GA may inhibit both the proteasome and MDM2, although these activities should be further validated. A α,β-unsaturated ketone structure was proposed to be able to target and inhibit the proteasome chymotrypsin-like β5 subunit^52^, and α,β-unsaturated ketone group of GA can form a covalent bond with a nucleophilic residue by the Michael reaction. Therefore, we tested the possible covalent docking between GA and the SH groups of cysteine residues in this proteasome subunit using molecular modeling studies. Indeed, our computational docking studies supported the potential of C10 atom of GA to bind to the Cys52 residue of the proteasome 20S β5 subunit (Supplementary Fig. 7A). In addition, we found that the C10 atom of GA demonstrates possible covalent binding to the Cys77 residue of MDM2 (Supplementary Fig. 7B). Thus, our molecular modeling studies suggest that GA can bind covalently with cysteine residues of the proteasome or MDM2 and thus, the proteasome and MDM2 may be included as potential targets of GA for the induction of paraptosis. Further detailed studies will be needed to test this hypothesis.

To selectively kill cancer cells, it is advantageous to exploit the differential characters of cancer cells and normal cells. Tumor cells often suffer from higher ER stress than normal cells due to the imbalance between a high metabolic demand and a limited protein-folding capacity^53^. When we examined whether GA-induced ER stress is differently modulated between breast cancer cells and normal cells, we found that GA treatment increased the levels of eIF2α phosphorylation and CHOP protein in MDA-MB 435S cells at its lower concentrations, compared to those in MCF-10A cells treated with GA (Supplementary Fig. 8A). Noxa upregulation was also more potently induced in GA-treated MDA-MB 435S cells, compared to that in GA-treated MCF-10A cells. Additionally, cancer cells exhibit greater oxidative stress than normal cells due to oncogenic stimulation, increased metabolic activity and mitochondrial malfunction^54^. To investigate the GA-induced functional change in breast cancer cells and normal cells, we analyzed the MMP using MTR together with Acridine Orange 10-Nonyl Bromide (NAO), a mitochondrial specific fluorescent probe. MMP loss was observed in MDA-MB 435S cells treated with GA for 16 h, but not in GA-treated MCF-10A cells (Supplementary Fig. 8B). These results suggest that GA may confer cancer cells much more stress to the ER and mitochondria than normal cells. In this respect, GA-mediated paraptosis may provide a two-pronged attack as a therapeutic strategy for selectively killing cancer cells that are already under the stress inherent to cancer.

## Materials and methods

### Chemicals and antibodies

Gambogic acid (GA) was purchased from Biorbyt (San Francisco, CA, UK). Manganese (III) tetrakis (4-benzoic acid) porphyrin chloride (MnTBAP) was from Calbiochem (EMD Millipore Corp., Billerica, MA, USA). MitoTracker-Red (MTR), propidium iodide (PI), 5-(and-6)-chloromethyl-2′,7′-dichlorofluorescein diacetate (CM-H_2_DCF-DA), and tetramethylrhodamine methyl ester (TMRM) along with secondary antibodies (rabbit IgG HRP (G-21234) and mouse IgG HRP (G-21040)) were from Molecular Probes (Eugene, OR, USA). The primary antibody against SDHA (ab14715) was from Abcam (Cambridge, MA, USA) and Noxa (OP180) was from Calbiochem. β-actin (sc-47778), ubiquitin (sc-8017), Mcl-1 (sc-819), ATF4 (sc-200) were from Santa Cruz Biotechnology (Santa Cruz, CA, USA). Phospho-SAPK/JNK (#9251), SAPK/JNK (#9252), phospho-ERK1/2 (#9101), ERK1/2 (#9102), TCF11/NRF1 (#8052), CHOP/GADD153 (#2895), phospho-eIF2α (#9721), and eIF2α (#9722) were from Cell Signaling Technology (Danvers, MA, USA). PDI (ADI-SPA-890-F) was form Enzo Life Science (Farmingdale, NY, USA). Other reagents were from Sigma-Aldrich (St Louis, MO, USA).

### Cell culture

MDA-MB 435S, MDA-MB 453, MDA-MB 468, MCF-10A, BxPC-3, and NCI-H460 cells were purchased from American Type Culture Collection (ATCC, Manassas, VA, USA). SNU-449 and SNU-668 cells were purchased from Korean Cell Line Bank (KCLB, Seoul, Korea). MDA-MB 435S cells were cultured in DMEM and MDA-MB 453, MDA-MB 468, BxPC-3, NCI-H460, SNU-449 and SNU-668 cells were cultured in RPMI-1640 medium supplemented with 10% fetal bovine serum and 1% antibiotics (GIBCO-BRL, Grand Island, NY, USA). MCF-10A cells were maintained in Mammary Epithelial Growth Medium (MEGM; Clonetics Corp., San Diego, CA, USA) supplemented with pituitary extract, insulin, human epidermal growth factor, hydrocortisone and choleratoxin (Calbiochem). Cells were incubated in 5% CO_2_ at 37 °C.

### Cell viability assay

Cells were cultured in 48-well plates and treated as indicated. The cells were then fixed with methanol/acetone (1:1) at −20 °C for 5 min, washed three times with PBS and stained with propidium iodide (PI; final concentration, 1 μg/ml) at room temperature for 10 min. The plates were imaged on an IncuCyte device (Essen Bioscience, Ann Arbor, MI, USA) and analyzed using the IncuCyte ZOOM 2016B software. The processing definition of the IncuCyte program was set to recognize attached (live) cells by their red-stained nuclei. The percentage of live cells was normalized to that of untreated control cells (100%).

### In vivo tumor growth inhibition assay

MDA-MB 435S cells were used to produce a xenograft tumor model in female BALB/c nude mice (nu/nu, 5 weeks old, Japan SLC, Hamamatsu, Japan). A suspension of 5 × 10^6^ cells in a 50 μl volume (saline) was subcutaneously injected into the right flank of mice. Tumors were grown for 3 weeks until average tumor volume reached 100~150 mm^3^. Mice were randomized into 3 groups (*n* = 4 per group), including vehicle (PBS containing 0.25% DMSO), 4 mg/kg GA and 8 mg/kg GA, and mice were received twice intraperitoneal (i.p.) injections of GA at the indicated concentrations (day 0 and day 2). Tumor size was measured three times a week for 2 weeks and tumor volume was calculated using the formula [*V* = (*L* x *W*^2^) x 0.5, where *V* = volume, *L* = length, and *W* = width]. All experiments were performed following the guidelines and regulations approved by the Institutional Animal Care and Use Committee of the Asan Institute for Life Science. On the 14th day, mice were sacrificed and the tumors were isolated, fixed in 4% paraformaldehyde and then embedded into paraffin. Sections of 5 μm were stained with H&E and the image on the tissue sections was observed and photographed by CMOS (Complementary metal-oxide-semiconductor) camera which is attached on K1-Fluo microscope (Nanoscope Systems, Daejeon, Korea).

### Examination of the morphologies of mitochondria and the ER employing the plasmids to specifically label the ER or mitochondria

Establishment of the stable cell lines expressing the fluorescence specifically in the ER lumen (YFP-ER cells) and the cell lines expressing the fluorescence specifically in mitochondria (YFP-Mito cells) were previously described^9,55^. Additionally, to label the ER membrane, MDA-MB 435S cells were transfected with the GFP-Sec61β (Addgene plasmid #15108) and the stable cell lines were selected with medium containing 500 μg/ml G418 (Calbiochem). Morphological changes of mitochondria or the ER were observed under confocal laser scanning microscope (K1-Fluo) using filter set (excitation band pass, 488 nm; emission band pass, 525/50).

### Immunoblot analyses and immunofluorescence microscopy

Immunoblot and immunofluorescence analysis was performed as described previously^9^. Images were acquired from Axiovert 200 M fluorescence microscope (Carl Zeiss, Oberkochen, Germany) using Zeiss filter sets #46 (excitation band pass, 500/20 nm; emission band pass, 535/30 nm), and #64HE (excitation band pass, 598/25 nm; emission band pass, 647/70 nm).

### Transmission electron microscopy

Cells were prefixed in Karnovsky’s solution (1% paraformaldehyde, 2% glutaraldehyde, 2 mM calcium chloride, 0.1 M cacodylate buffer, pH 7.4) for 2 h and washed with cacodylate buffer. Post-fixing was carried out in 1% osmium tetroxide and 1.5% potassium ferrocyanide for 1 h. After dehydration with 50–100% alcohol, the cells were embedded in Poly/Bed 812 resin (Pelco, Redding, CA), polymerized, and observed under electron microscope (EM 902 A, Carl Zeiss).

### Measurement of ROS generation

Treated cells were incubated with 10 μM of CM-H_2_DCF-DA for 30 min at 37 °C, and subjected for the fluorescence microscopy. Images were acquired from Axiovert 200 M fluorescence microscope using Zeiss filter sets #46 (excitation band pass, 500/20 nm; emission band pass, 535/30 nm).

### Analysis of mitochondrial membrane potential

To analyze mitochondrial membrane potential (Δ*ψ*), cells (8 × 10^4^ cells) cultured in 12-well plates were treated with 1 μM GA or 20 μM carbonyl cyanide *m*-chlorophenyl hydrazine (CCCP) for the indicated time points and incubated for 30 min at 37 °C with 200 nM TMRM or 100 nM MTR. After washing with PBS, cells were observed under confocal laser scanning microscope (K1-Fluo). For fluorescence-activated cell sorting analysis, TMRM stained cells were washed PBS, detached from dishes, and subjected for using a FACScan system (BD Biosciences, SanJose, CA, USA).

### In vitro reactions of GA with GSH or NAC, and LC-MS/MS analysis

GA was adjusted to 100 μM in 0.1 mL methanol and then mixed with 0.1 mL of either 50 mM GSH or 50 mM NAC. After 3 h incubation at 40 °C, the reaction was quenched by the addition of 20-fold ice-cold methanol. For monitoring of GA alone and its formation of adducts with NAC or GSH, 1-μL aliquot of the mixture was directly injected into an Agilent 6470 Triple Quad LC-MS/MS system (Agilent, Wilmington, DE, USA) coupled to an Agilent 1260 HPLC system. Chromatographic separation was performed on a Synergi Polar RP (4 μm, 2.0 mm i.d. x 150 mm, Phenomenex, Torrance, CA, USA) using a mobile phase that comprised water and methanol (15:85, v/v) containing 0.1% formic acid. Mass spectra were recorded by electrospray ionization in the positive mode.

### Detoxification of GA with *N*-acetylcysteine (NAC)

To test the detoxification of GA by NAC, aliquots of serum-free DMEM containing 1 μM GA and different concentrations of NAC were pre-incubated at room temperature for the indicated time points, and then incubated with MDA-MB 435S cell cultures for 24 h. To examine the effect of simultaneous treatment of GA and NAC, cells were treated with 1 μM GA and increasing concentrations of NAC without pre-incubation. Subsequently, cell viability was assessed by PI staining using IncuCyte.

### Fluorescence labeling of protein thiol groups by dibromobimane (dBrB) assay

MDA-MB 435S cells plated in 60 mm plates were treated as indicated, harvested, resuspended in PBS, and sonicated. A part of samples was used for the measurement of the protein concentration using Lowry-based assay and the rest of samples were immediately reacted with 1.5 N perchloric acid and incubated for 5 minutes on ice to precipitate the proteins. The samples were centrifuged at 14,000 x g for 10 minutes, and the pelleted proteins were solubilized with 0.1 M NaOH and neutralized using 0.5 M Tris-HCl. The prepared proteins (1 μg) were mixed with 1 μM dibromobimane and incubated for 40 min at 37 °C. Dibromobimane-bound protein-SH groups were measured in a fluorescence multiplate reader (Synergy H1 Hybrid Multi-Mode reader; BioTek, Winooski, VT, USA) at Ex/Em 393/477 nm. Fluorescence was normalized by the total protein levels and expressed as percentage of protein-SH levels compared to that from the untreated group.

### Statistical analysis

All data are presented as mean ± SEM (standard error of the mean) or ± SD (standard deviation) from at least three separate experiments. To perform statistical analysis, GraphPad Prism (GraphPad Software Inc, Sandiego, CA) was used. Normality of data was assessed by Kolmogorov–Smirnov testes and equal variance using Bartlett’s test. For normal distribution, statistical differences were determined using an analysis of variance (ANOVA) followed by followed by Bonferroni multiple comparison test. If the data were not normally distributed, Kruskal–Wallis test was performed followed by Dunn’s test.

## Supplementary information

Supplementary Information
