# Supplementary material for: Gambogic acid triggers vacuolization-associated cell death in cancer cells via disruption of thiol proteostasis
Source: Cell Death Dis. 2019 Feb 22;10(3):187. doi: 10.1038/s41419-019-1360-4 (PMC6385239; doi:10.1038/s41419-019-1360-4)
Supplement: Supplementary file 1 — Supplementary Information [file 41419_2019_1360_MOESM1_ESM.docx]

**Supplementary Information**

**Supplementary Material and Methods**

**Chemicals**

Necrostatin-1, 3-methyladenine (3-MA), and bafilomycin A1 were purchased from Sigma-Aldrich (St Louis, MO, USA). z-VAD-fmk was from R&D systems (Minneapolis, MN, USA). Bortezomib was from Selleckchem (Houston, TX, USA) and nutlin-3 was from TOCRIS (Avonmouth, Bristol, UK). Acridine Orange 10-Nonyl Bromide (NAO), Calcein-acetoxymethyl ester (calcein-AM) and ethidium homodimer-1 (EthD-1) were from Molecular Probes (Eugene, OR, USA).

**Cell viability assay (Live & Dead assay)**

Cells were cultured in 24-well plates and treated with the indicated concentrations of GA for 24 h. For measurement of cellular viability, 2 μM calcein-AM, a green fluorescent indicator of the intracellular esterase activity of cells, and 4 μM EthD-1, a red fluorescent indicator of membrane damaged (dead) cells, were added to each well, and the plates were incubated for 5 min in 5% CO_2_ at 37°C. Cells were then observed under a fluorescence microscope (Axiovert 200M; Carl Zeiss, Oberkochen, Germany) equipped with Zeiss filter sets #46 (excitation band pass, 500/20 nm; emission band pass, 535/30 nm), and #64HE (excitation band pass, 598/25 nm; emission band pass, 647/70nm). Viable cells, corresponding to those that exclusively exhibited green fluorescence, were counted in five fields per well at 200 × magnification. Only exclusively green cells were counted as live because bicolored (green and red) cells cannot be unambiguously assigned to live or dead groups. The percentage of live cells (Live %), calculated as green cells/(green + red + bicolored cells), was normalized to that of untreated control cells (100%).

**Computational modeling**

To understand the mode through which GA binds to the proteasome or MDM2, we performed a docking study using the covalent docking protocol in the Schrödinger Suite 2018-1 (Glide v7.8, Schrödinger, LLC, New York, NY). The X-ray crystal structure of MDM2 (PDB code 5C5A) and proteasome 20S chymotrypsin β5 (PDB code 5LF3) were obtained from the Protein Data Bank (http://www.rcsb.org). GA was minimized using an OPLS_2005 force field with a dielectric constant of 80.0, as applied using the MacroModel v11.9 program. The binding modes of GA (ball and stick model) and the protein surface model were visualized using Discovery Studio 2018 (Biovia, Discovery Studio Modeling Environment, Dassault Systèmes, San Diego, CA).

**Morphological examination of mitochondria**

To observe morphological changes of mitochondria, MDA-MB 435S or MCF-10A cells (8 x 10 ^4^ cells) cultured in 12-well plates were treated with 1 μM GA for the indicated time points and incubated for 30 min at 37 °C with 100 nM NAO. After washing with PBS, cells were observed under confocal laser scanning microscope (K1-Fluo).

**Supplementary Figures**

**
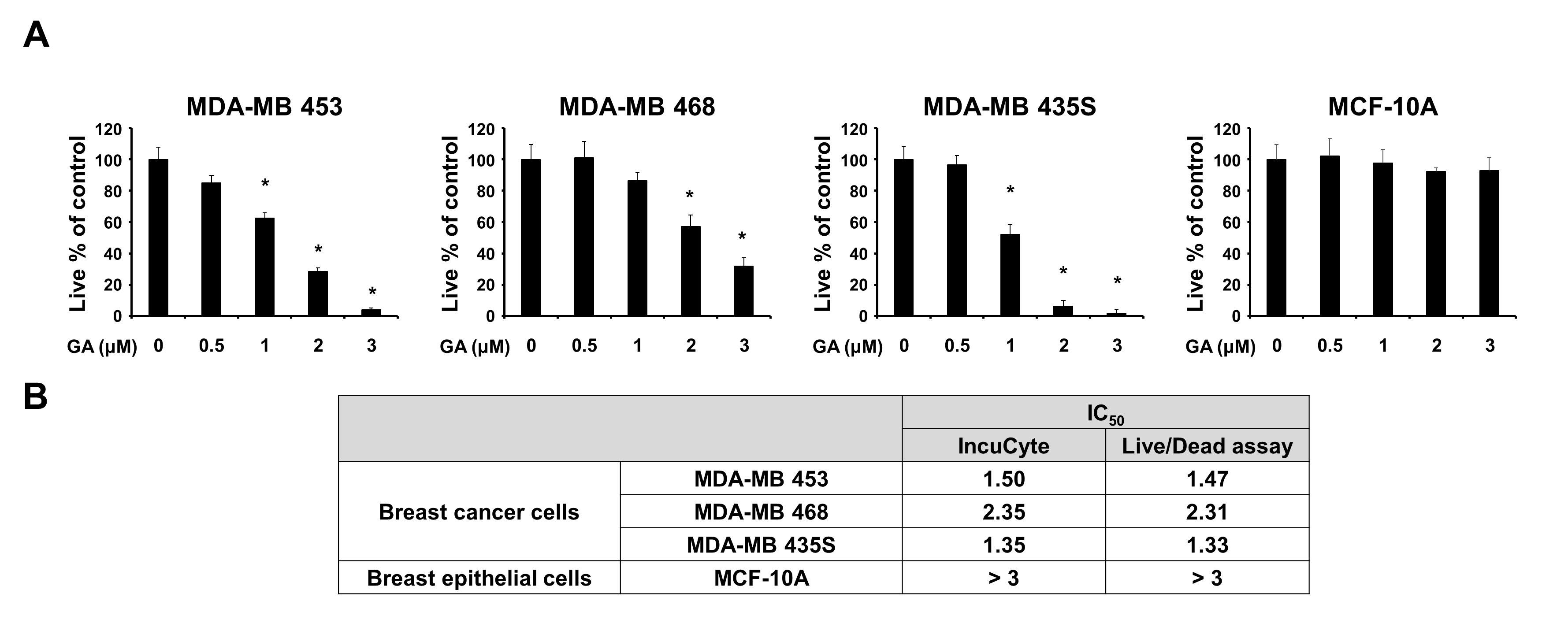
**

**Supplementary Fig. 1 GA reduces cell viability.**

A Cells were treated with the indicated concentrations of GA for 24 h. Cellular viability was assessed using Live & Dead kit as described in Supplementary Materials and Methods. Data represent the means ± SD (*n* = 7). Statistical significance was determined using one-way ANOVA followed by Bonferroni’s *post hoc* tests. **p* < 0.01 vs. untreated control. **B** IC_50_s were calculated using GraphPad Prism.

**
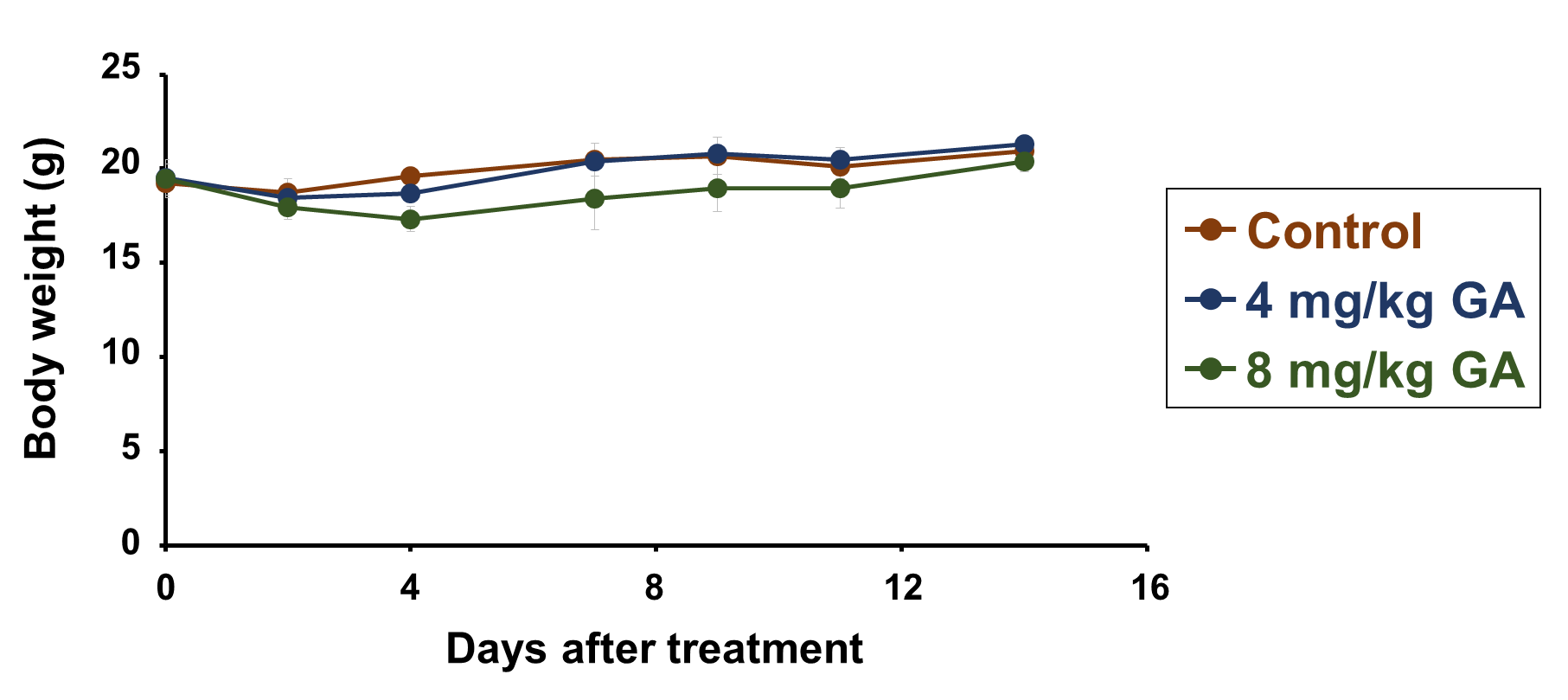
**

**Supplementary Fig. 2** **GA does not induce a significant loss of body weight in mice with MDA-MB 435S xenograft tumor.**

The body weight of mice was measured every 2-3 days after the beginning of vehicle or GA injection.

**
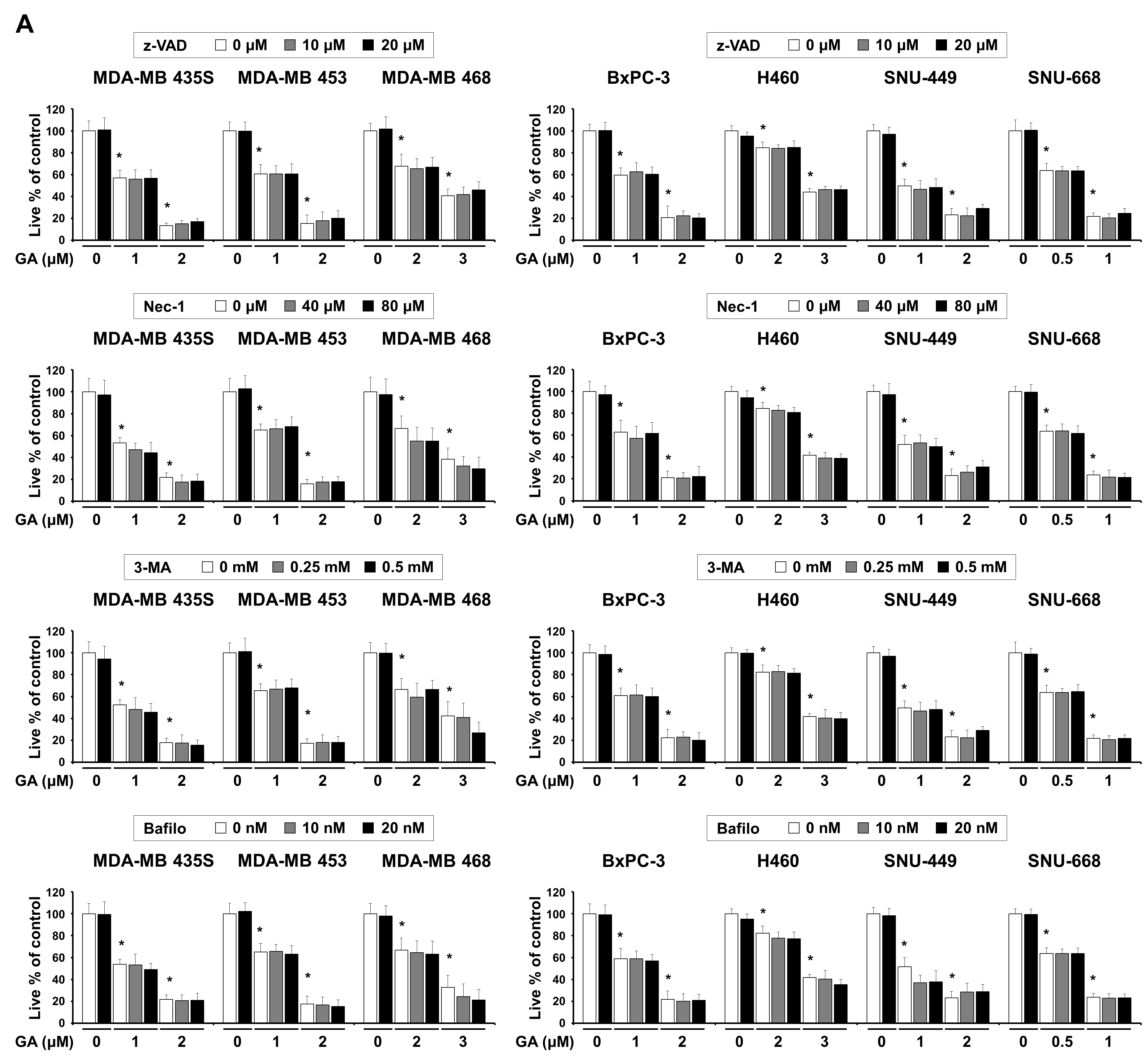
**

**
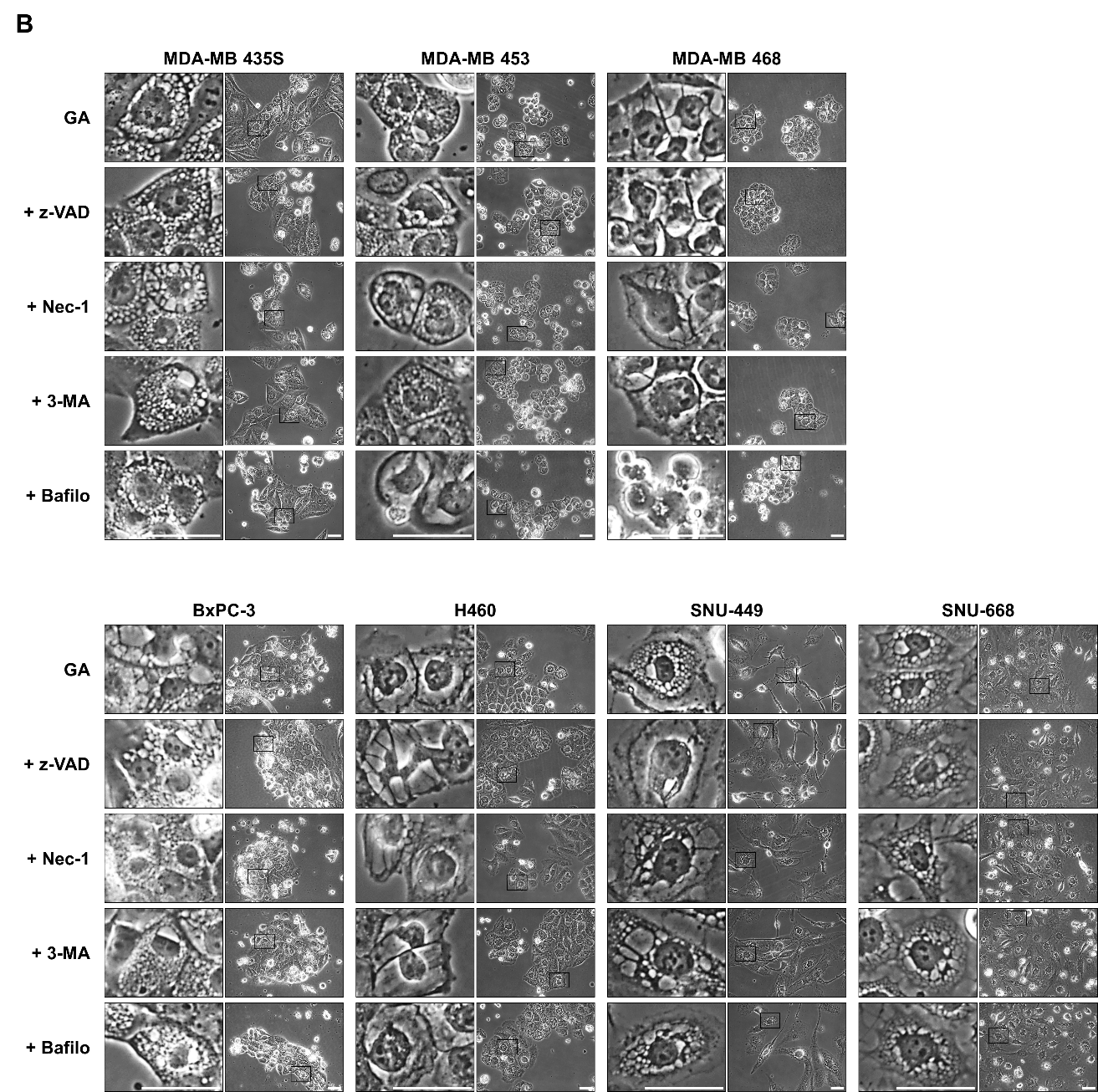
**

**Supplementary Fig. 3 GA induces cell death accompanied by vacuolation, which is not dependent on apoptosis, necroptosis or autophagy, in various types of cancer cells.**

**A** Cells were pretreated with the indicated concentrations of z-VAD-fmk (z-VAD), necrostatin-1 (Nec-1), 3-methyladenine (3-MA), or bafilomycin A1 (Bafilo) and further treated with GA for 24 h. Cellular viability was assessed using IncuCyte. Data represent the means ± SEM (*n* = 3). One-way ANOVA and Bonferroni’s *post hoc* test. **p* < 0.01 vs. untreated control. **B** Cells were pretreated with 20 μM z-VAD, 80 μM Nec-1, 0.5 mM 3-MA, or 20 nM Bafilo and further treated with GA (0.5 μM for SNU-668; 1 μM for MDA-MB 435S, BxPC-3 and SNU-449, 2 μM for MDA-MB 453; 3 μM for MDA-MB 468 and NCI-H460 cells) for 12 h. Cells were observed by phase-contrast microscopy. Bars, 40 μm.

**
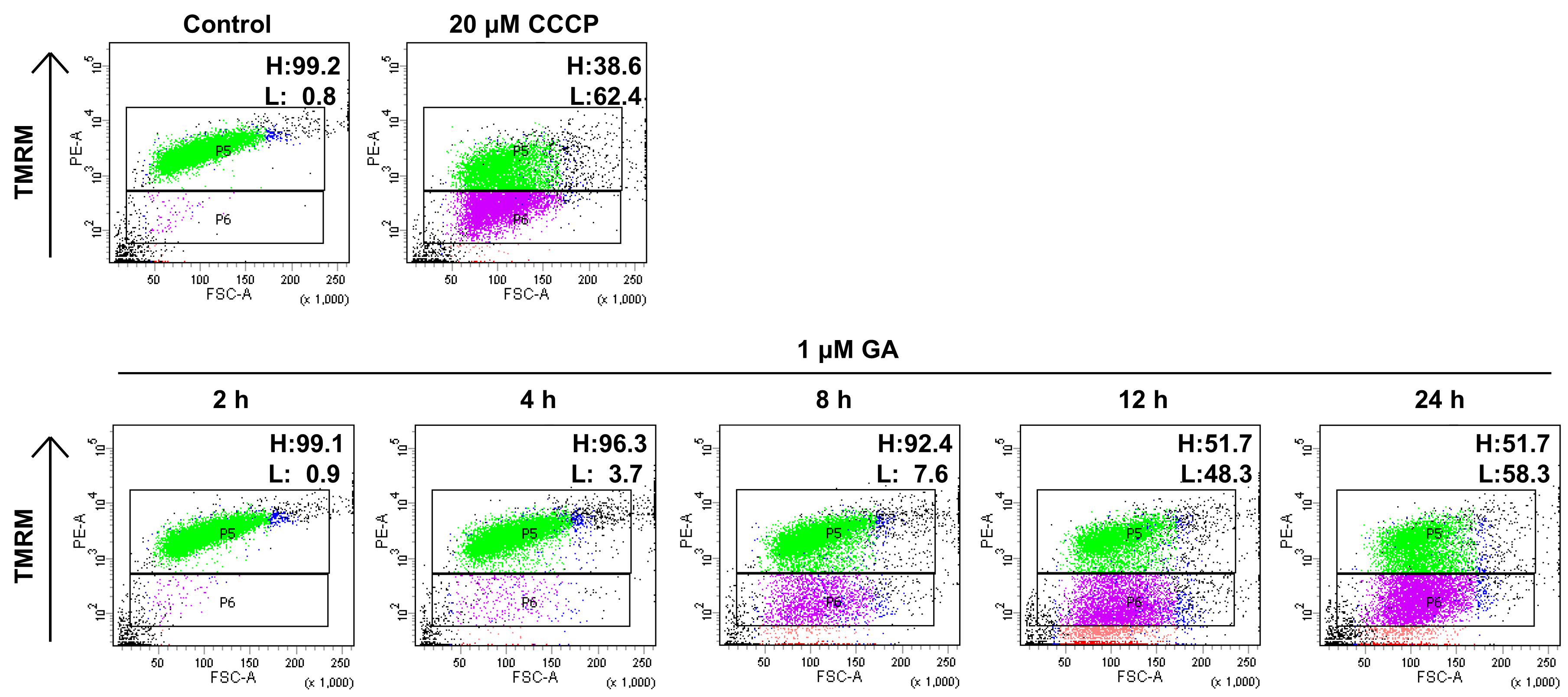
**

**Supplementary Fig. 4 MMP is decreased in cells treated with GA.**

MDA-MB 435S cells treated with 1 μM GA for the indicated time points or 20 μM CCCP for 12 h were incubated with TMRM and subjected for FACS. Percentages of high Δψ cells (TMRM-positive cells in P5) and low Δψ cells (TMRM-negative cells in P6) were denoted as H and L, respectively.

**
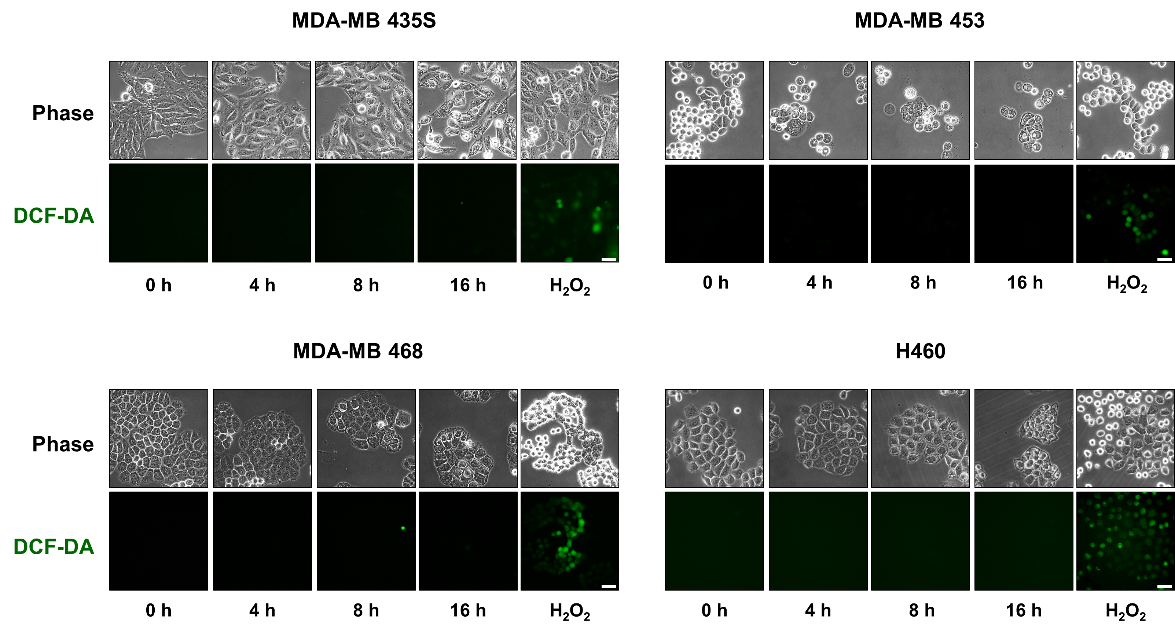
**

**Supplementary Fig. 5 GA does not markedly increase ROS levels during GA-induced paraptosis.**

Cells were treated with GA (1 μM for MDA-MB 435S, 2 μM for MDA-MB 453, 3 μM for MDA-MB 468, H460 cells) for the indicated time points and or treated with 5 mM H_2_O_2_ for 10 min. Treated cells were incubated with CM-H_2_DCF-DA (DCF-DA) and subjected for the fluorescence microscopy. Bars, 40 μm.


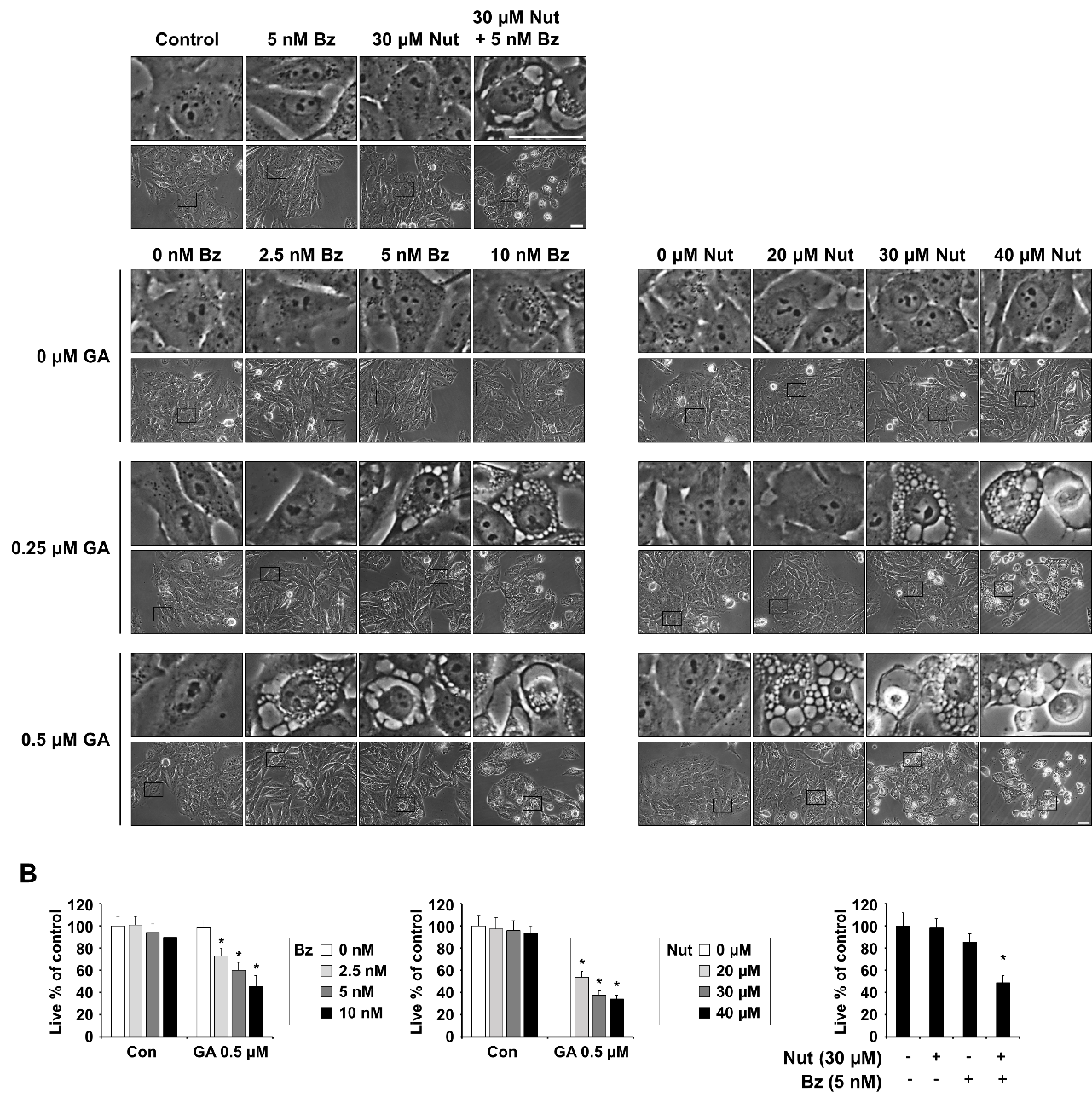


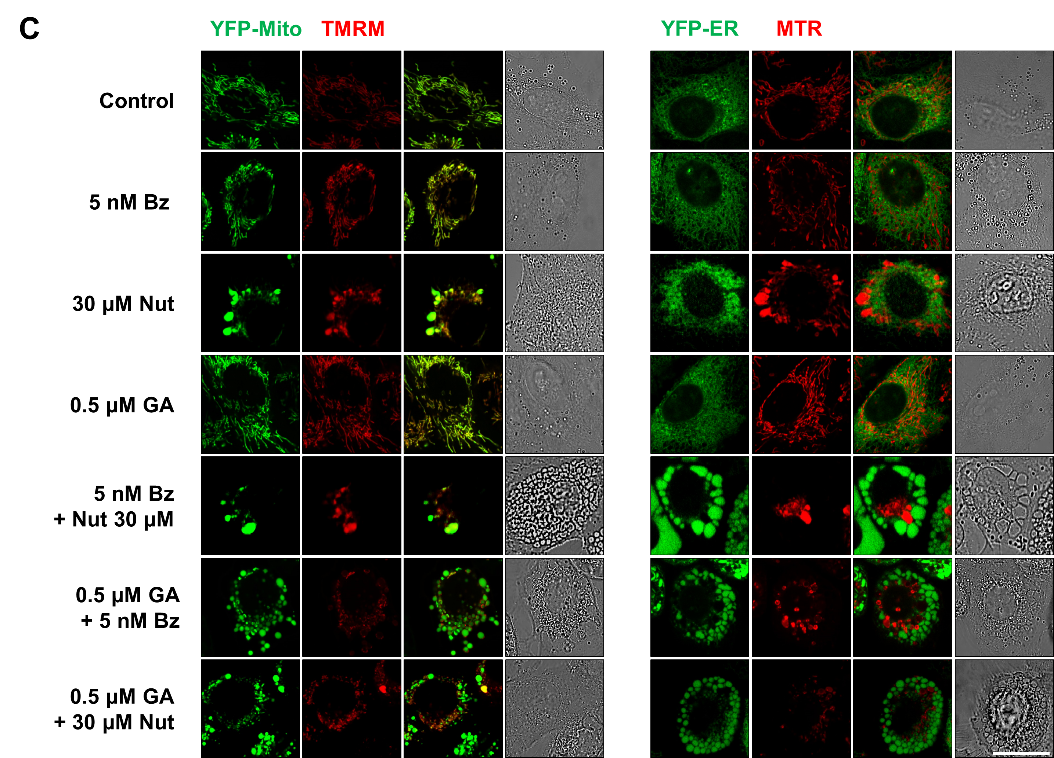


**Supplementary Fig. 6 Bortezomib or nutlin-3 sensitizes cancer cells to GA-mediated paraptosis.**

**A, B** MDA-MB 435S cells were treated with bortezomib (Bz) plus nutlin-3 (Nut), GA plus Bz or GA plus Nut at the indicated concentrations for 24 h. (**A**) Cells were observed under the phase-contrast microscope. Bars, 40 μm. (**B**) Cell viability was assessed using IncuCyte. Data represent the means ± SD. Statistical significance was determined using one-way ANOVA followed by Bonferroni’s *post hoc* tests. **p* < 0.05 vs. untreated control. **C**YFP-Mito cells were treated with the chemicals as indicated for 12 h and incubated with 200 nM TMRM for 20 min. YFP-ER cells were treated with the chemicals as indicated for 12 h and incubated with 100 nM MTR for 10 min. Cells were observed by confocal microscopy. Bars, 20 μm.


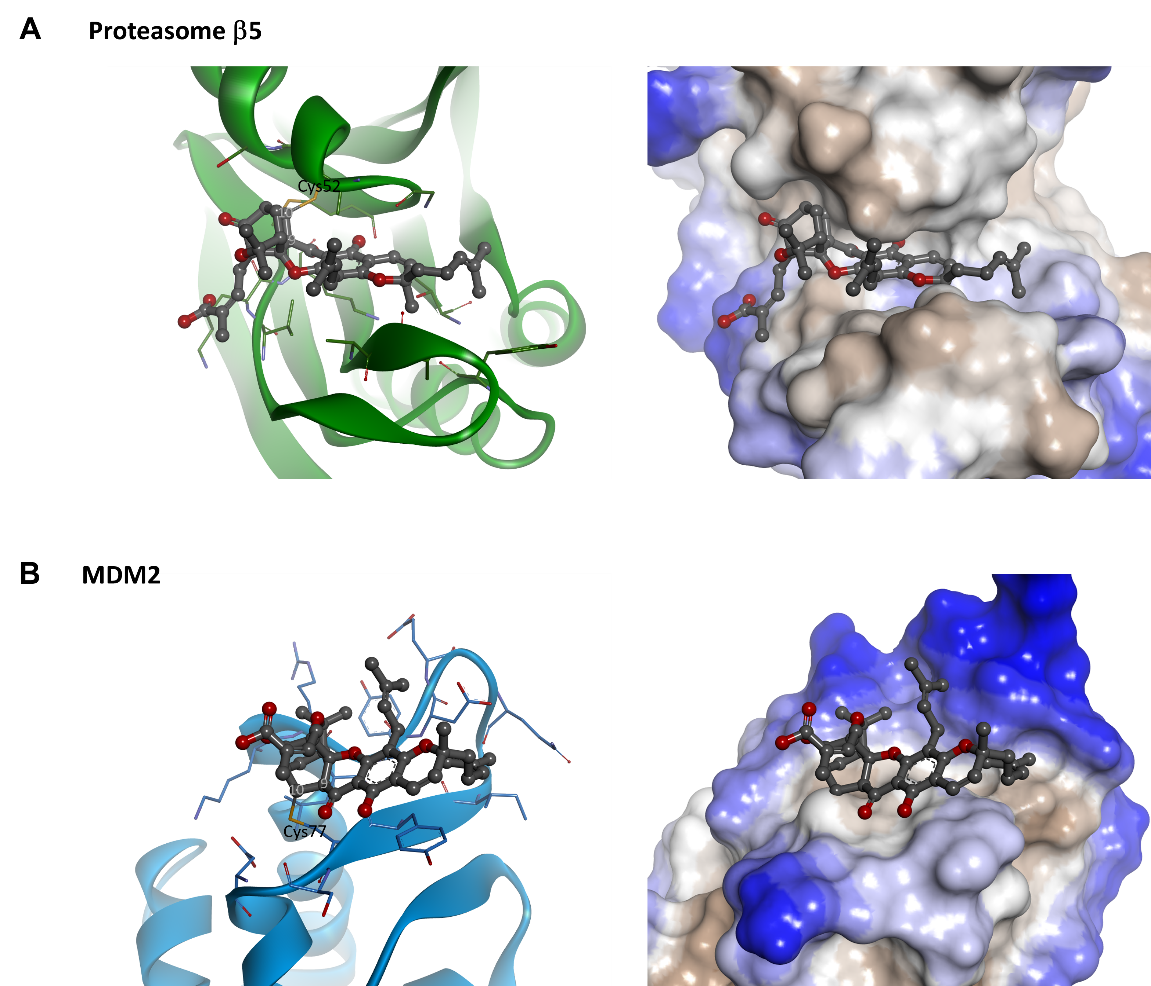


**Supplementary Fig. 7 Computational modeling to predict the binding mode of GA to the proteasome β5 subunit or MDM2.**

Left panels show predicted binding modes of GA in the active site of proteins and right panels exhibit the surface models of the active site bound to GA. For clarity, the cysteine residue is labeled using its 3-letter amino acid code and the surface model is colored by hydrophobicity (blue for hydrophilic; brown for hydrophobic). **A** The potential covalent bonding between the C10 atom of GA and the Cys52 residue of the proteasome 20S β5 subunit. **B** The potential covalent bonding between the C10 atom of GA and the Cys77 residue of MDM2.


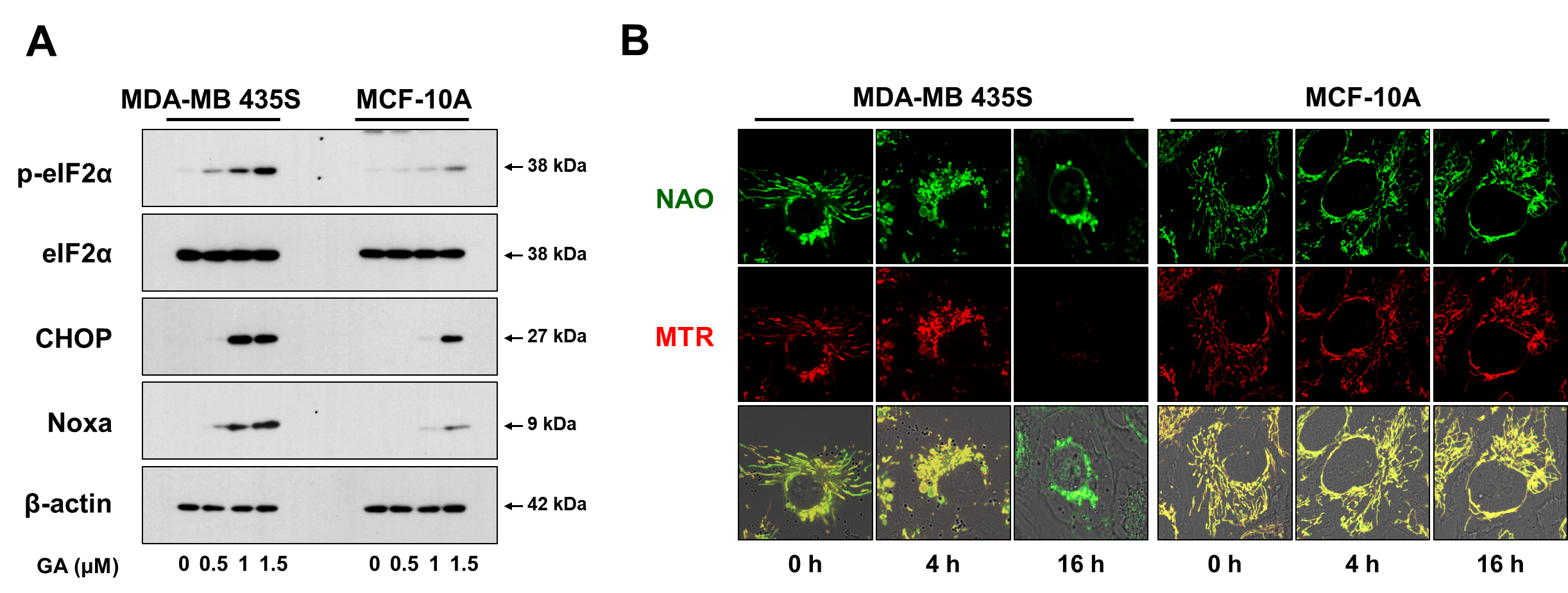


**Supplementary Fig. 8** **GA-induced ER stress and MMP loss are differently regulated in MDA-MB 435S and MCF-10A cells.**

**A** MDA-MB 435S and MCF-10A cells were treated with the indicated concentrations of GA for 8 h and Western blotting of phospho-eIF2α, CHOP, and Noxa were performed, with β-actin as a loading control. **B** MDA-MB 435 or MCF-10A cells treated with 1 μM GA for the indicated time durations were incubated with 100 nM MTR and 100 nM NAO for 30 min at 37°C. After washing with PBS, cells were observed by confocal microscopy.


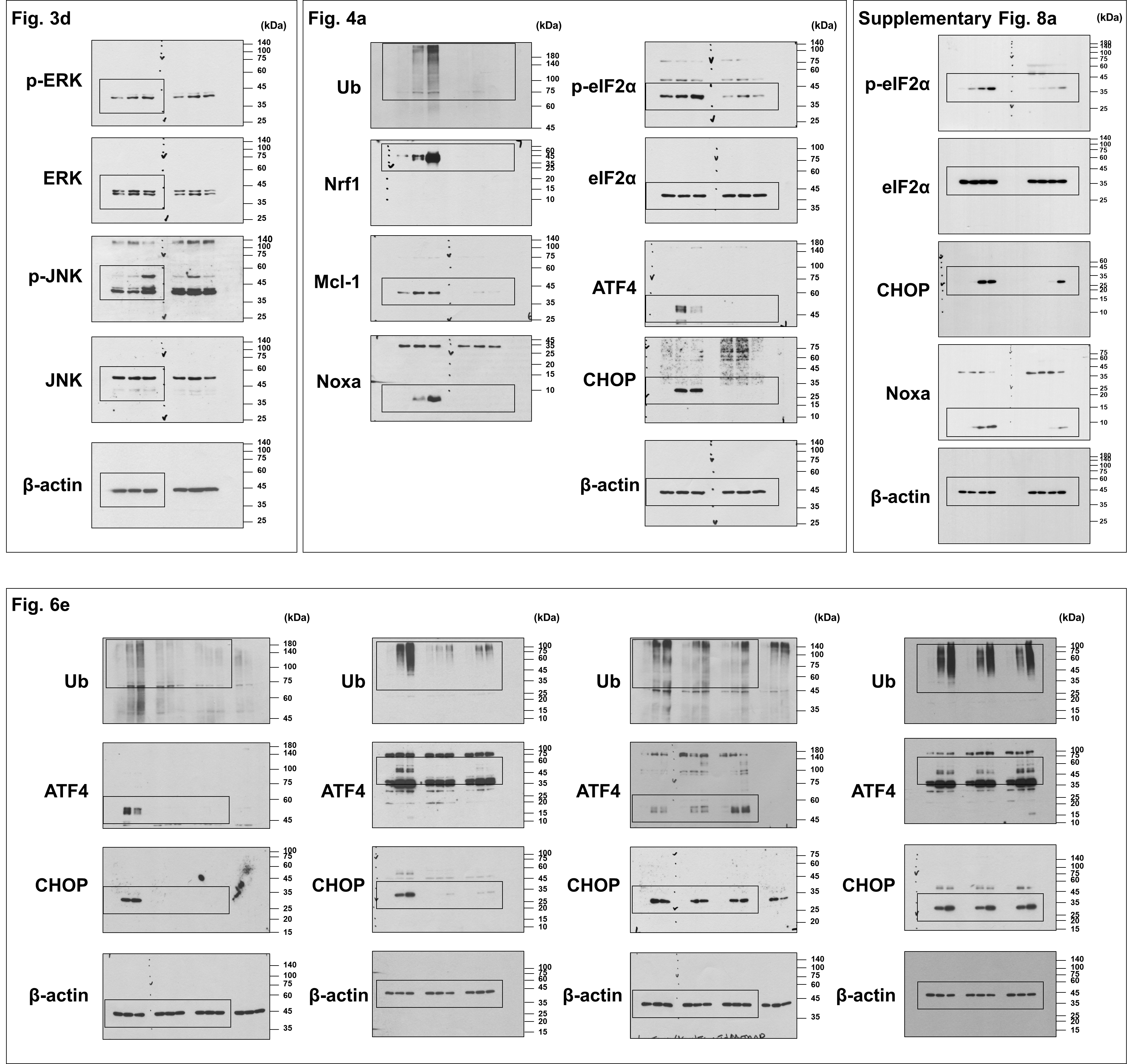


**Supplementary Fig. 9 The full scan for all the western blot images in this study.**

The black lined-boxes on the western data indicate the cropped images used in Fig. 3D, Fig. 4A, Fig. 6E and Supplementary Fig. 8A.
